# Differential SOD2 and GSTZ1 profiles contribute to contrasting dental pulp stem cell susceptibilities to oxidative damage and premature senescence

**DOI:** 10.1186/s13287-021-02209-9

**Published:** 2021-02-17

**Authors:** Nadia Y. A. Alaidaroos, Amr Alraies, Rachel J. Waddington, Alastair J. Sloan, Ryan Moseley

**Affiliations:** 1 Regenerative Biology Group, Oral and Biomedical Sciences, School of Dentistry, Cardiff Institute of Tissue Engineering and Repair (CITER), College of Biomedical and Life Sciences, Cardiff University, Cardiff, CF14 4XY UK; 2 Melbourne Dental School, Faculty of Medicine, Dentistry and Health Sciences, The University of Melbourne, Melbourne, Australia

**Keywords:** Dental pulp stem cells, Heterogeneity, Oxidative stress, Oxidative damage, Premature senescence, SOD2, GSTZ1

## Abstract

**Background:**

Dental pulp stem cells (DPSCs) are increasingly being advocated as viable cell sources for regenerative medicine-based therapies. However, significant heterogeneity in DPSC expansion and multi-potency capabilities are well-established, attributed to contrasting telomere profiles and susceptibilities to replicative senescence. As DPSCs possess negligible human telomerase (hTERT) expression, we examined whether intrinsic differences in the susceptibilities of DPSC sub-populations to oxidative stress-induced biomolecular damage and premature senescence further contributed to this heterogeneity, via differential enzymic antioxidant capabilities between DPSCs.

**Methods:**

DPSCs were isolated from human third molars by differential fibronectin adhesion, and positive mesenchymal (CD73/CD90/CD105) and negative hematopoietic (CD45) stem cell marker expression confirmed. Isolated sub-populations were expanded in H_2_O_2_ (0–200 μM) and established as high or low proliferative DPSCs, based on population doublings (PDs) and senescence (telomere lengths, SA-β-galactosidase, p53/p16^INK4a^/p21^waf1^/hTERT) marker detection. The impact of DPSC expansion on mesenchymal, embryonic, and neural crest marker expression was assessed, as were the susceptibilities of high and low proliferative DPSCs to oxidative DNA and protein damage by immunocytochemistry. Expression profiles for superoxide dismutases (SODs), catalase, and glutathione-related antioxidants were further compared between DPSC sub-populations by qRT-PCR, Western blotting and activity assays.

**Results:**

High proliferative DPSCs underwent > 80PDs in culture and resisted H_2_O_2−_induced senescence (50–76PDs). In contrast, low proliferative sub-populations exhibited accelerated senescence (4–32PDs), even in untreated controls (11-34PDs). While telomere lengths were largely unaffected, certain stem cell marker expression declined with H_2_O_2_ treatment and expansion. Elevated senescence susceptibilities in low proliferative DPSC (2–10PDs) were accompanied by increased oxidative damage, absent in high proliferative DPSCs until 45–60PDs. Increased SOD2/glutathione S-transferase ζ1 (GSTZ1) expression and SOD activities were identified in high proliferative DPSCs (10–25PDs), which declined during expansion. Low proliferative DPSCs (2–10PDs) exhibited inferior SOD, catalase and glutathione-related antioxidant expression/activities.

**Conclusions:**

Significant variations exist in the susceptibilities of DPSC sub-populations to oxidative damage and premature senescence, contributed to by differential SOD2 and GSTZ1 profiles which maintain senescence-resistance/stemness properties in high proliferative DPSCs. Identification of superior antioxidant properties in high proliferative DPSCs enhances our understanding of DPSC biology and senescence, which may be exploited for selective sub-population screening/isolation from dental pulp tissues for regenerative medicine-based applications.

**Supplementary Information:**

The online version contains supplementary material available at 10.1186/s13287-021-02209-9.

## Background

Dental pulp stem cells (DPSCs) are increasingly being advocated as a viable stem cell source in the development of regenerative medicine-based therapies, based on their accessibility, self-renewal, clonogenicity, and multi-potency [1–3]. However, a drawback associated with DPSC-based therapy development is their significant heterogeneity within dental pulp tissues, with individual clones demonstrating major differences in proliferation and differentiation capabilities [4–6]. Consequently, despite heterogeneous DPSC populations achieving > 120 population doublings (PDs) in vitro, only 20% of purified DPSCs are capable of proliferating > 20PDs. Such issues are confounded by DPSCs being proposed to exist within distinct niches within dental pulp tissues (sub-odontoblast layer, pulpal vasculature and central pulp) [7], which increases their complexity regarding the origins and regenerative characteristics of individual DPSC sub-populations. Such features have major implications for successful DPSC exploitation, as a significant limitation of mesenchymal stem cell (MSC)-based therapies is the extensive in vitro expansion necessary to produce sufficient cell numbers for clinical use. Consequently, cell expansion eventually leads to proliferative decline and replicative (telomere-dependent) senescence, characterized by progressive telomere shortening, inhibition of G_1_-S phase transition and permanent growth arrest. This is associated with the loss of telomeric TTAGGG repeats, positive senescence-associated (SA)-β-galactosidase staining, and increased tumor suppressor (p53 and retinoblastoma protein, pRb) and cyclin-dependent kinase inhibitor (p21^waf1^ and p16^INK4a^) gene expression [8–10]. Such events are recognized to significantly alter MSC genotype and phenotype, leading to impaired regenerative properties and disrupted local tissue micro-environment signaling mechanisms, through the secretome associated with the senescence-associated secretory phenotype (SASP) [10, 11].

Despite significant differences in the ex vivo expansion capabilities of individual DPSC sub-populations, only recently have studies investigated such variations in proliferative capabilities and senescence susceptibilities on the multi-potency and other properties of different DPSC sub-populations [12, 13]. High proliferative DPSCs are reported to achieve > 80PDs, whereas low proliferating DPSCs only complete < 40PDs before senescence, correlating with DPSCs with high proliferative capacities possessing longer telomeres than less proliferative sub-populations. Low proliferative DPSC senescence was also associated with the loss of stem cell marker characteristics and impaired osteogenic/chondrogenic differentiation, in favor of adipogenesis. In contrast, high proliferative DPSCs retained multi-potency capabilities, only demonstrating impaired differentiation following prolonged in vitro expansion (> 60PDs). As most studies have reported no or negligible reverse transcriptase, human telomerase catalytic subunit (hTERT) expression in human DPSCs [12, 14–16], hTERT is unlikely to be responsible for maintaining telomere integrity and the proliferative/multi-potency capabilities of high proliferative DPSCs. Therefore, other intrinsic mechanisms may account for differences in telomere lengths, proliferation rates, and differentiation capabilities between high and low proliferative DPSC sub-populations.

Oxidative stress is a well-established mediator of telomere-independent, premature senescence, including in MSCs [8, 10, 17]. However, although replicative and oxidative stress-induced senescence share common characteristics, premature senescence is not associated with extensive telomere shortening. Reactive oxygen species (ROS) are generated via numerous cellular mechanisms, with low ROS levels playing important roles in regulating MSC signaling and the maintenance of stemness and multi-potent differentiation properties [18, 19]. Although endogenous enzymic and non-enzymic antioxidant defense mechanisms counteract ROS accumulation and regulate cellular redox homeostasis, excessive ROS exposure is implicated in causing indiscriminate oxidative damage to biomolecules, such as DNA, proteins, and lipids [20–22], and accelerating premature senescence [8, 10, 17]. Differences in cellular susceptibilities to oxidative stress-induced biomolecular damage and premature senescence are often associated with contrasting enzymic antioxidant profiles between cell types, such as superoxide dismutases (SODs), catalase, and glutathione-metabolizing enzymes, including glutathione peroxidases (GPXs), S-transferases (GSTs), reductases (GSRs), and synthetases (GSSs) [23–26]. Indeed, imbalance between ROS production and cellular enzymic antioxidant capacities are strongly correlated with increased susceptibilities to oxidative stress-induced damage and senescence [27–37].

In light of the evidence attributing superior endogenous enzymic antioxidant capabilities with cellular resistance to oxidative stress, we investigated whether similar differences in DPSC susceptibilities to oxidative stress-induced biomolecular damage and premature senescence existed, due to differential enzymic antioxidant capabilities between DPSC sub-populations. Consequently, this is the first study to confirm inherent differences in enzymic antioxidant expression profiles between high and low proliferative DPSC sub-populations. Such SOD2 and glutathione S-transferase ζ1 (GSTZ1) adaptations would contribute to the protection of high proliferative DPSCs from oxidative damage and senescence, thereby helping explain DPSC sub-population heterogeneity overall.

## Materials and methods

### Stem cell isolation and culture under oxidative stress conditions

Human DPSCs were isolated from third molar teeth collected from patients (all female, age 18–30 years) undergoing orthodontic extractions at the School of Dentistry, Cardiff University, UK. Teeth were collected in accordance with the Declaration of Helsinki (2013), with informed patient consent and ethical approval by the South East Wales Research Ethics Committee of the National Research Ethics Service (NRES), UK.

Single-cell suspensions of dental pulp tissues were obtained, with DPSCs preferentially selected and isolated from cell suspensions by differential fibronectin adhesion assay [12]. DPSC colonies (≥ 32 cells) were subsequently harvested and maintained at 37 °C/5% CO_2_ in α-modified Minimum Essential Medium (αMEM), containing ribonucleosides and deoxyribonucleosides, supplemented with 4 mM l-glutamine, 100 U/mL penicillin G sodium, 0.1 μg/mL streptomycin sulfate, 0.25 μg/mL amphotericin, 20% fetal calf serum (FCS) (all ThermoFisher Scientific, Paisley, UK), and 100 μM l-ascorbate 2-phosphate (Sigma, Poole, UK). Once established, DPSC sub-populations were seeded at 5000 cells/cm^2^ in T-75 flasks and expanded at 37 °C/5% CO_2_ in αMEM medium under continuous exposure to sub-lethal doses of exogenous H_2_O_2_ (0, 50 μM, 100 μM, or 200 μM; ThermoFisher Scientific), throughout their proliferative lifespans to senescence. Medium was changed every 2 days.

### Population doubling analysis

On reaching 80–90% confluence, DPSCs expanded with or without H_2_O_2_ (0–200 μM), were treated with StemPro**®** Accutase (Thermo Fisher Scientific) and PD rates calculated from cell counts throughout their proliferative lifespans, as previously described [12, 38, 39]. Cumulative PDs were subsequently plotted against time in culture, with the onset of cellular senescence confirmed when DPSCs underwent < 0.5 PDs/week [12, 38].

### Telomere restriction fragment (TRF) length analysis

At selected PDs throughout their proliferative lifespans, DPSCs expanded with or without H_2_O_2_ (0–200 μM) were maintained in 6-well plates as above, until 80–90% confluent. Following DNA purification [12], telomere length analyses were performed using the TeloTAGGG Telomere Restriction Fragment (TRF) Length Assay Kit (Roche, Welwyn Garden City, UK), per the manufacturer’s instructions. A digoxigenin (DIG)-labeled molecular weight marker (kb, *in Kit*) and positive DIG-labeled control DNA sample (CTRL, *in Kit*) were also included. Mean telomere lengths were calculated from Southern blot images via ImageJ® Software [12].

### Senescence-associated β-galactosidase staining

DPSCs expanded with or without H_2_O_2_ (0–200 μM) were seeded in 6-well plates at 5000 cells/cm^2^. DPSC senescence was assessed by the presence of SA-β-galactosidase staining using a Senescence Cells Histochemical Staining Kit (Sigma), as previously described [12, 38].

### Senescence- and stem cell-related gene expression analysis

Reverse transcription polymerase chain reactions (RT-PCR) were employed for the analysis of senescence (p53, p16^INK4a^, p21^waf1^, hTERT) and stem cell (CD73, CD90, CD105, CD45, CD117, CD146, CD166, CD271, BMI-1, Nanog, Oct4, Slug, SSEA4) marker gene expression. DPSCs expanded with or without H_2_O_2_ (0–200 μM) were maintained in 6-well plates as above, until 80–90% confluent. Total RNA extraction, cDNA generation, and PCR reactions were performed as previously described [12, 39], using primer sequences described in Supplementary Table S1 (Primer Design, Southampton, UK), with a β-actin housekeeping gene. Total human RNA (Thermo Fisher Scientific) was used as a positive control for all genes analyzed, while cDNA replacement with nuclease-free water served as negative controls. PCR products and 100 bp DNA ladders (Promega, Southampton, UK) were separated on 2% agarose gels in 1× Tris-acetate-EDTA buffer. Images were captured under UV light and analyzed, as previously described [12, 39].

### Immuno-detection of oxidative stress-induced biomarkers

DPSCs expanded with or without H_2_O_2_ (0–200 μM) were assessed for the presence of oxidative DNA and protein biomarker levels, by immunocytochemistry using 8-well chamber slides (VWR International, Lutterworth, UK). Oxidative DNA damage, in the form of 8-hydroxy-deoxy-guanosine (8-OHdG) levels [21], was detected using fluorometric OxyDNA Assay Kits (Merck Millipore, Watford, UK). Oxidative protein damage (in the form of protein carbonyl content [22]), was detected using fluorometric OxyICC™ Oxidized Protein Detection Kits (Merck Millipore). Control wells were included for each Kit, consisting of phosphate-buffered saline (PBS), instead of fluorescent conjugates. Chamber slides were subsequently mounted using FluorSave Reagent (Merck Millipore) and viewed using a Leica Dialux 20 Fluorescent Microscope (Leica Microsystems, Milton Keynes, UK). Images were captured using HCImage acquisition and analysis software (Hamamatsu Corporation, Sewickley, PA, USA).

### Enzymic antioxidant gene expression

Quantitative real-time polymerase chain reaction (qRT-PCR) analysis of enzymic antioxidant (SOD1, SOD2, SOD3, CAT1, GPX1, GPX2, GPX3, GPX4, GPX5, GSR, GSS, GSTZ1) gene expression was performed as previously described [40]. DPSCs at selected PDs throughout their proliferative lifespans were cultured, RNA extracted, and cDNA synthesized, as above. cDNA amplification was performed using the Applied Biosystems™ ViiA™ 7 Real-Time PCR System and TaqMan® primers (ThermoFisher Scientific, Supplementary Table S2), according to the manufacturer’s protocols. qRT-PCR was performed MicroAmp™ Fast Optical 96-Well Reaction Plates (ThermoFisher Scientific), per the manufacturer’s instructions. Relative fold changes in enzymic antioxidant gene expression (RQ) were calculated using the 2^–ΔΔCt^ method [41], normalized versus an 18S rRNA housekeeping gene.

### Western blot analysis

DPSCs expanded with or without H_2_O_2_ (0–200 μM) were maintained in T-75 flasks as above, until 80–90% confluent. Cultures were harvested with RIPA buffer (400 μL/flask, ThermoFisher Scientific), containing cOmplete™ Protease Inhibitor Cocktail (Roche), according to manufacturer’s instructions. Extracts were sonicated and protein concentration quantified (Pierce® BCA Protein Assay Kit, ThermoFisher Scientific). Protein samples (10 μg) were separated by sodium dodecyl sulfate-polyacrylamide gel electrophoresis (SDS-PAGE) on pre-formed 4–15% TGX™ gels (Mini-Protean® Tetra Cell System; Bio-Rad, Hemel Hempstead, UK); and electroblotting onto polyvinylidene difluoride membranes (Hybond™-P; ThermoFisher Scientific), using a Mini Trans-Blot® Electrophoretic Transfer Cell (Bio-Rad), per the manufacturer’s instructions. Membranes were blocked with 5% semi-skimmed milk/1% Tween 20 in Tris-buffered saline (TBS), for 1 h at room temperature. Membranes were immuno-probed with primary antibodies (Abcam, Cambridge, UK), specific to SOD1 (ab16831, 1:1000); SOD2 (ab13534, 1:1000); SOD3 (ab21974, 1:1000), and GSTZ1 (ab153995, 1:500). Normalized protein loading was confirmed by β-actin Loading Control (ab8227, 1:20,000, Abcam). Immuno-probing occurred in 5% semi-skimmed milk/1% Tween 20, at 4 °C overnight or room temperature for 1 h. Membranes were washed (× 3) in 1% TBS-Tween and incubated in HRP-conjugated swine anti-rabbit secondary antibody (P039901-2, 1:5000, Dako, Ely, UK), in 5% semi-skimmed milk/1% Tween 20, for 1 h at room temperature. Membranes were washed (× 3) in 1% TBS-Tween and TBS. Membranes were incubated in ECL™ Prime Detection Reagent (VWR International) and autoradiographic films (Hyperfilm™-ECL, Thermo Fisher Scientific) developed, per manufacturer’s instructions. Immunoblot images were captured and densitometry performed using ImageJ® Software, with untreated controls at each respective time-point representing 1.0-fold.

### Enzymic antioxidant activity analysis

DPSCs expanded with or without H_2_O_2_ (0–200 μM) were harvested for assessment of total SOD, catalase, and GPX activities, per manufacturer’s instructions. Total SOD activities were determined using SOD Activity Colorimetric Assay Kits (Abcam). Total catalase activities were determined using Catalase Specific Activity Assay Kits (Abcam). Total GPX activities were determined using Glutathione Peroxidase Assay Kits (Cambridge Bioscience, Cambridge, UK). Sample absorbance values were read spectrophotometrically using a using a FLUOstar® Omega Plate Reader (BMG Labtech, Aylesbury, UK) and total SOD, catalase, and GPX activities in cell extracts determined versus SOD (*in Kit*), catalase (from human erythrocytes, Sigma), and GPX (from human erythrocytes, Sigma) standard curves.

### Statistical analysis

Each experiment was performed on *n* = 3 independent occasions. Statistical analyses were performed using GraphPad Prism (GraphPad Software, La Jolla, CA, USA). Data were expressed as mean ± standard error of mean (SEM) and statistically compared using analysis of variance (ANOVA), with post hoc Tukey test. Significance was considered at *p* < 0.05.

## Results

### DPSC population doublings under oxidative stress conditions

Several DPSC sub-populations were successfully isolated and characterized from 3 individual patient donors (patients A, C, and D). As significant variations in proliferative capacity and susceptibilities to replicative senescence have previously been identified within different DPSC sub-populations [12], initial studies assessed the effects of continual sub-culture under oxidative stress (0–200 μM H_2_O_2_) conditions on PDs throughout their proliferative lifespans to senescence for individual DPSC sub-populations. Overall, PDs and proliferative capacities showed marked variations in DPSC susceptibilities to premature senescence, with PD differences irrespective of which patient DPSCs were derived.

**Fig. 1:**
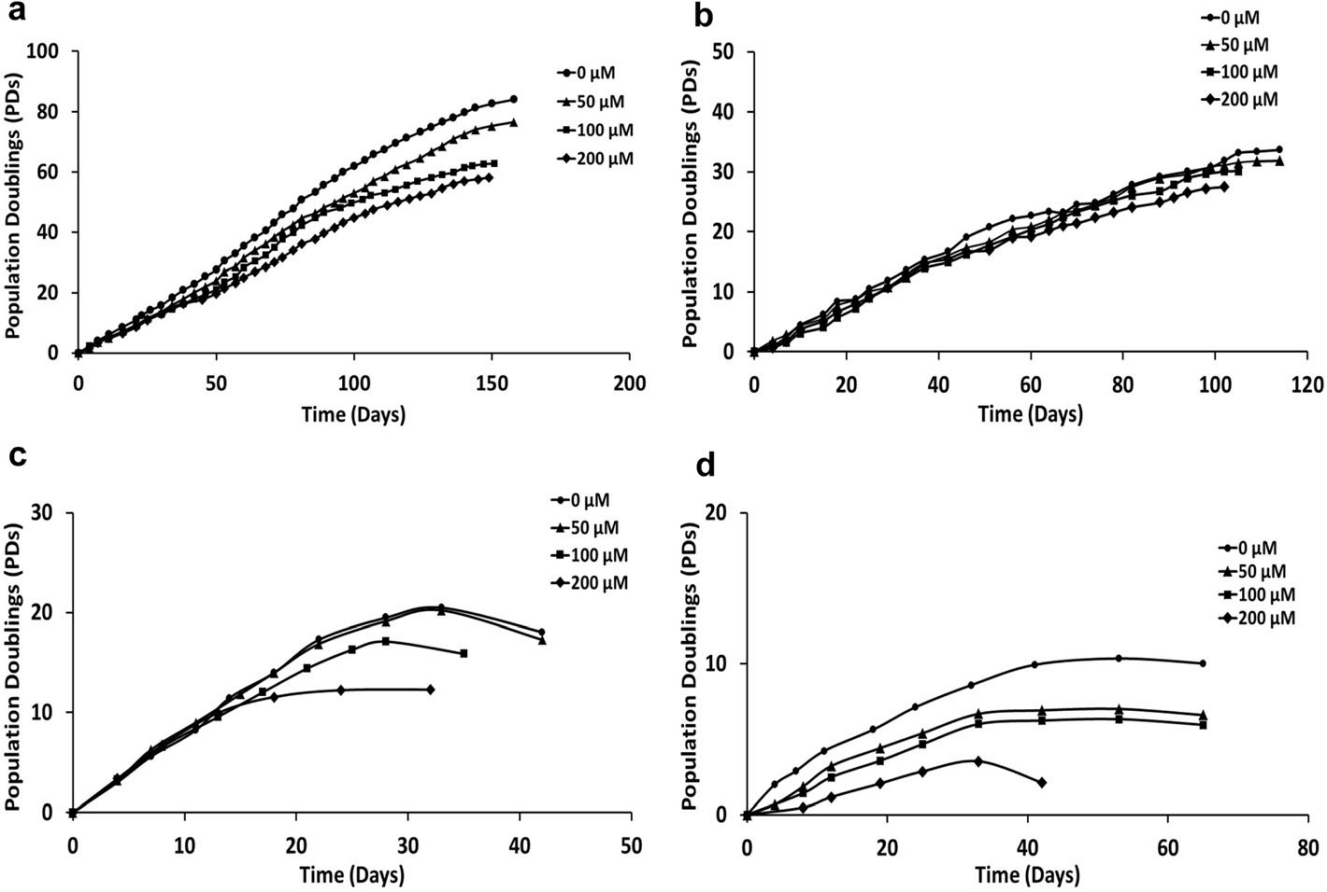
DPSC population doublings (PDs) during extended culture with or without exogenous H_2_O_2_ (50–200 μM) treatment. **a** High proliferative sub-population, A1 (patient A), exhibited high resistance to oxidative stress-induced premature senescence, achieving 50–76PDs with H_2_O_2_ treatment, compared to untreated controls (> 80PDs). **b** Low proliferative sub-population, A2 (patient A), only achieved 27–32PDs with H_2_O_2_ treatment, compared to untreated controls (34PDs). **c** Low proliferative sub-population, C3 (patient C), only achieved 12–19PDs with H_2_O_2_ treatment, compared to untreated controls (20PDs). **d** Low proliferative sub-population, D4 (patient D), only achieved 4–7PDs with H_2_O_2_ treatment, compared to untreated controls (11PDs)

DPSC populations, such as A1 (Patient A), achieved the highest PDs upon H_2_O_2_ treatment and greatest resistance to H_2_O_2_-induced senescence, compared to untreated controls (> 80PDs). These sub-populations reached 50–76PDs following continual treatment with 50–200 μM H_2_O_2_ over 145–162 days in culture, prior to senescence (Fig. 1a). In contrast, low proliferative DPSCs, such as A2, C3, and D4 (patients A, C, and D, respectively), all exhibited earlier inductions of premature senescence, even in untreated controls. DPSC population, A2, was only capable of achieving 27–32PDs with 50–200 μM H_2_O_2_ over 98–120 days in culture, compared to untreated controls (34PDs, Fig. 1b). Furthermore, populations C3 and D4 only accomplished 12–19PDs (25–43 days in culture) and 4–7PDs (30–40 days in culture) with 50–200 μM H_2_O_2_, versus their untreated counterparts (20PDs and 11PDs, Fig. 1c, d, respectively).

### DPSC telomere lengths

**Fig. 2:**
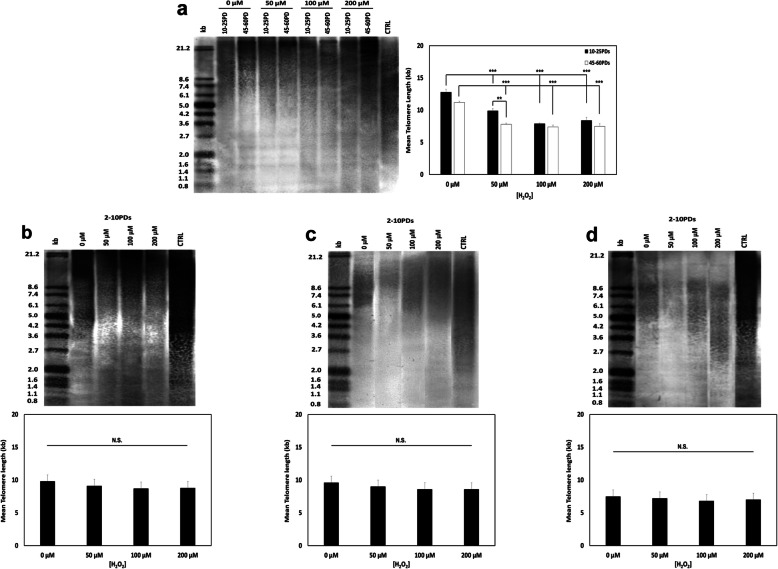
DPSC telomere lengths during extended culture with or without exogenous H_2_O_2_ (50–200 μM) treatment. Representative images of TRF analysis (determined by Southern blotting) and mean telomere lengths (ImageJ® analysis), determined for **a** high proliferative sub-population, A1 (10–25PDs and 40–65PDs) and **b–d** low proliferative sub-populations A2 (2–10PDs), C3 (2–10PDs), and D4 (2–10PDs). Left- and right-hand lanes represent separated DIG-labeled telomere length standards (kb, *in Kit*). CTRL represent telomere length positive control (*in Kit*). *N* = 3, values in graphs represent the mean ± SEM. ***p < 0.001. N.S. = Non-significant

Despite contrasting telomere lengths being implicated in the proliferative and multi-potency heterogeneity between DPSC sub-populations [12], numerous studies have reported the limited impact of oxidative stress on telomere shortening during premature senescence [8, 10, 17]. Consequently, we next examined telomere lengths in the DPSC sub-populations and whether oxidative stress conditions influenced telomere lengths during culture expansion. Mean telomere lengths for DPSCs sub-populations, A2, C3, and D4, with and without H_2_O_2_ (50–200 μM), were measured at 2–10PDs, with quantification of telomere lengths in DPSCs, A1, at 10–25PDs and 45–60PDs. Mean telomere lengths varied between DPSC sub-populations, with untreated high proliferative DPSC sub-population, A1, possessing longer telomeres (12.8 kb) at 10–25PDs (Fig. 2a), versus low proliferative DPSCs, A2 (9.8 kb, Fig. 2b), C3 (9.6 kb, Fig. 2c), and D4 (7.5 kb, Fig. 2d), at 2–10PDs. High proliferative sub-population, A1, exhibited significant 2–5 kb reductions in telomere length with increasing H_2_O_2_ treatment at 10–25PDs and 45–60PDs (all *p* < 0.001, Fig. 2a). Untreated A1 demonstrated minor reductions (< 2 kb) in telomere lengths (11.2 kb) with culture expansion between 10–25PDs and 45–60PDs, with similar length reductions at 45–60PDs with 100–200 μM H_2_O_2_ (all *p* > 0.05). However, significant telomere length reductions were shown between 10–25PDs and 45–60PDs with 50 μM H_2_O_2_ (*p* < 0.01). In contrast, low proliferative DPSCs, A2, C3, and D4, demonstrated no significant decreases in telomere lengths (≤ 1 kb), with increasing H_2_O_2_ treatment at 2–10PDs (all *p* > 0.05, Fig. 2b–d, respectively).

### DPSC senescence-related marker detection

**Fig. 3:**
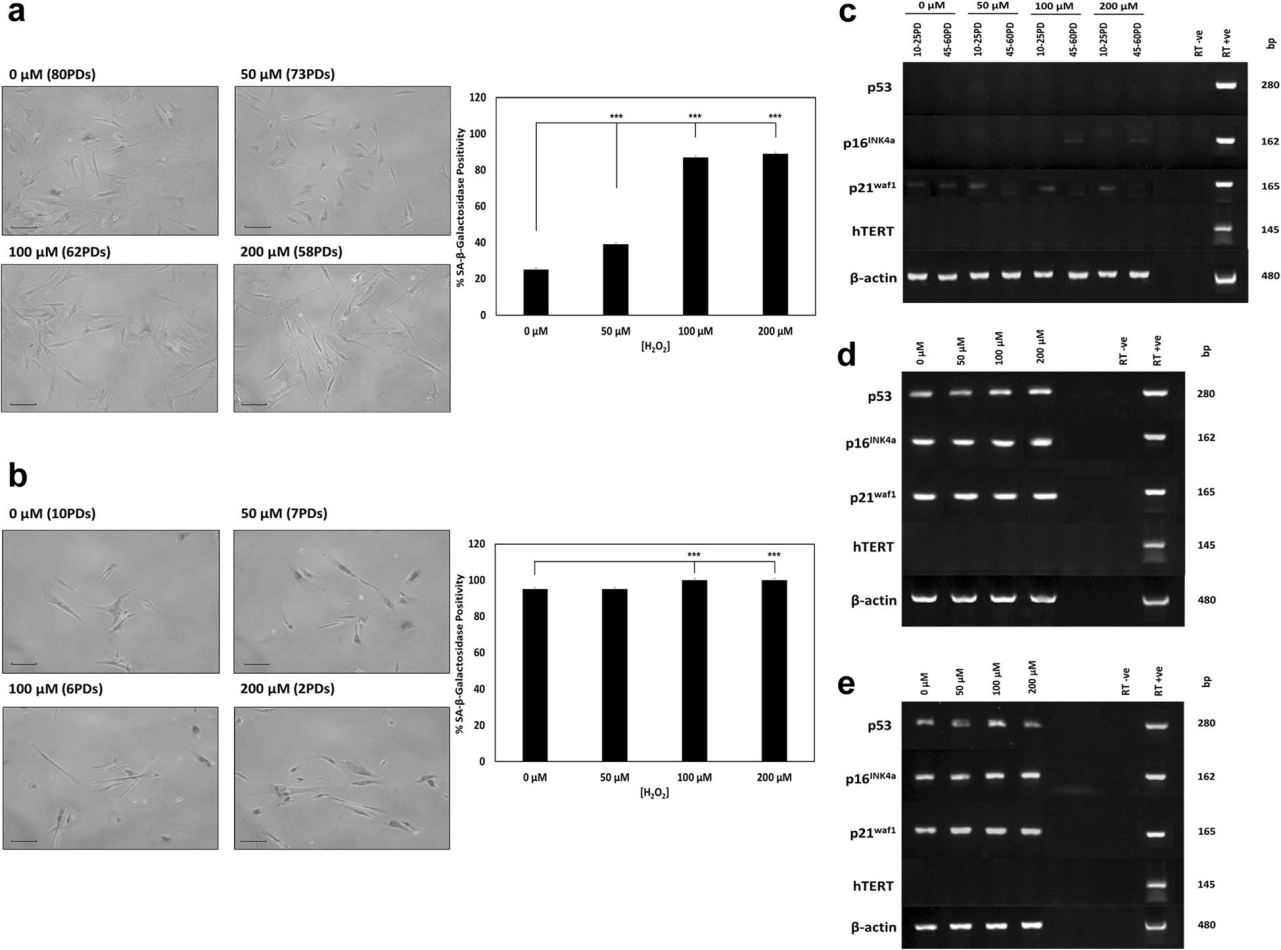
Senescence-related marker detection during extended DPSC culture with or without exogenous H_2_O_2_ (50–200 μM) treatment. **a, b** Representative SA-β-galactosidase microscopy images and % positively stained cell calculations, for high proliferative sub-population, A1 (58–80PDs) and low proliferative sub-population, D4 (2–10PDs). Scale bar 100 μm, × 100 magnification. *N* = 3, values represent the mean ± SEM. ***p < 0.001 versus untreated DPSC controls. Characterization of senescence marker (p53, p16^INK4a^, p21^waf1^, hTERT) expression for **c** high proliferative sub-population, A1 (10–25PDs and 40–65PDs), **d, e** low proliferative DPSC sub-populations C3 (2–10PDs) and D4 (2–10PDs). β-actin was used as the housekeeping gene. Right-hand lanes represent separate total human RNA-positive controls, water and RT-negative controls. bp = base pairs

Numerous studies have reported the increased detection of senescence markers in MSC populations due to oxidative stress-induced premature senescence [10, 17]. Further analyses of SA-β-galactosidase-positive staining and cellular senescence-related gene expression confirmed that DPSC sub-population, A1, exhibited the strongest resistance to oxidative stress-induced premature senescence overall. SA-β-galactosidase positivity was only particularly evident in A1 after 58PDs with H_2_O_2_ treatment (Fig. 3a), particularly with 100–200 μM H_2_O_2_ treatments (89% and 87% positivity, respectively). Significantly less SA-β-galactosidase positivity was detectable in untreated A1 controls, even following 80PDs in culture (25%, *p* < 0.001). In contrast, low proliferative DPSC sub-populations, A2, C3, and D4, demonstrated increased SA-β-galactosidase detection at much earlier PDs, especially with increasing H_2_O_2_ exposure (Figs. 3b and S1). DPSC sub-population, A2, demonstrated ≥ 86% positivity at 28PDs and 26PDs, with C3 exhibiting ≥ 69% positivity at 16PDs and 12PDs, following 100–200 μM H_2_O_2_ treatments (all *p* < 0.001 versus untreated controls). Similarly, untreated and 50 μM H_2_O_2_-treated sub-population, D4, displayed 95% positive SA-β-galactosidase staining at 10PDs and 7PDs (Fig. 3b, *p* > 0.05), and 100% positive staining with 100–200 μM H_2_O_2_ treatments (6PDs and 2PDs, respectively, *p* < 0.001 versus untreated controls).

Cellular senescence-related gene (p53, p16^INK4a^, p21^waf1^, hTERT) expression showed that all DPSCs were negative for hTERT expression (Fig. 3c–e). High proliferative sub-population, A1, displayed undetectable p53 and p16^INK4a^ expression at 10–25PDs, irrespective of H_2_O_2_ treatment. However, low level p21^waf1^ expression by A1 was apparent at 10–25PDs and 45–60PDs, independent of H_2_O_2_ treatment (Fig. 3c). Low level p16^INK4a^ expression was also detectable for A1, but only at 45–60PDs with 100–200 μM H_2_O_2_. Both low proliferative DPSC sub-populations, C3 and D4, demonstrated strong p53, p21^waf1^ and p16^INK4a^ expression at 2–10PDs, independent of H_2_O_2_ treatment (Fig. 3d, e, respectively), although low proliferative DPSC sub-population, A2, demonstrated low p53, p16^INK4a^, and p21^waf1^ expression at 2–10PDs, irrespective of H_2_O_2_ treatment (Fig. S2).

### DPSC stem cell marker detection

**Fig. 4:**
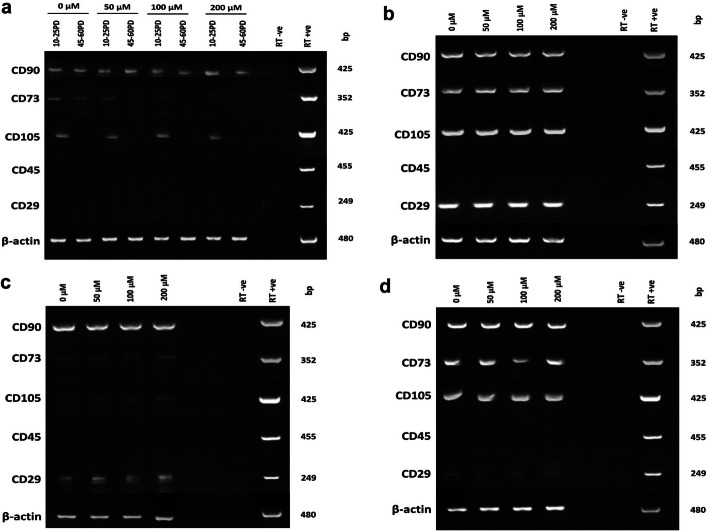
Mesenchymal and hematopoietic stem cell marker expression during extended DPSC culture with or without exogenous H_2_O_2_ (50–200 μM) treatment. **a** High proliferative sub-population, A1 (10–25PDs and 40–65PDs), and **b–d** low proliferative sub-populations A2 (2–10PDs), C3 (2–10PDs), and D4 (2–10PDs). β-actin was used as the housekeeping gene. Right-hand lanes represent separate total human RNA-positive controls, water, and RT-negative controls. bp = base pairs. *N* = 3

As DPSC senescence is commonly associated with the loss of stem cell marker expression [12, 39], RT-PCR analysis was performed to determine whether increasing H_2_O_2_ treatment accelerated the loss of stem cell characteristics in DPSCs. All DPSC sub-populations showed varying positive gene expression for MSC markers, CD73, CD90, and CD105 (Fig. 4a–d). In contrast, hematopoietic stem cell marker, CD45, was undetectable in all DPSCs. CD90 expression was largely retained in all DPSC sub-populations through culture expansion, irrespective of H_2_O_2_ treatment. However, CD73 and CD105 expression by high proliferative sub-population, A1, showed declined detection at 40–65PDs, dependently and independently of H_2_O_2_ treatment (Fig. 4a). In contrast, reductions in CD73 and CD105 expression were less evident in low proliferative DPSCs, A2, C3, and D4 (Fig. 4b–d, respectively).

**Fig. 5:**
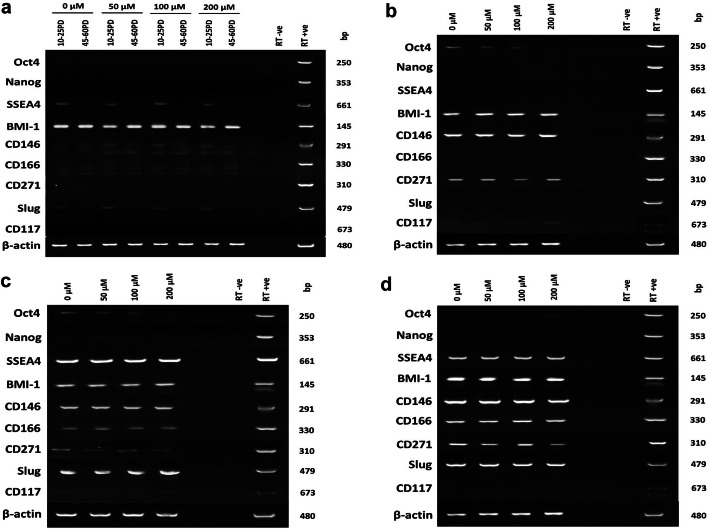
Mesenchymal, embryonic and neural crest stem cell marker expression during extended DPSC culture with or without exogenous H_2_O_2_ (50–200 μM) treatment. **a** High proliferative sub-population, A1 (10–25PDs and 40–65PDs), and **b–d** low proliferative sub-populations A2 (2–10PDs), C3 (2–10PDs), and D4 (2–10PDs). β-actin was used as the housekeeping gene. Right-hand lanes represent separate total human RNA-positive controls, water, and RT-negative controls. bp = base pairs. *N* = 3

MSC multi-potency markers, CD29 (Fig. 4a–d), CD146, and CD271 (Fig. 5a-d), were only particularly evident in low proliferative sub-populations, A2, C3, and D4, with expression being unaffected by H_2_O_2_ treatment, except CD271. Similarly, stem cell differentiation regulator, CD166, expression was only detectable with low proliferative DPSCs, C3 and D4 (Fig. 5a–d). However, all DPSC sub-populations exhibited positive expression for self-renewal/multi-potent adult stem cell marker, BMI-1 (Fig. 5a–d). Analysis of DPSC embryonic/neural crest marker expression showed that Oct4 was absent in high proliferative sub-population, A1 (Fig. 5a), but expressed by all low proliferative DPSCs analyzed (Fig. 5b–d). Other embryonic markers, SSEA4 and Slug, were positively expressed in high proliferative sub-population, A1 (Fig. 5a), and low proliferative DPSCs, C3 and D4 (Fig. 5c, d, respectively) only. Increased H_2_O_2_ treatment and culture expansion reduced SSEA4 and Slug expression in high proliferative DPSCs and Oct4 in low proliferative DPSCs. Negligible pluripotency (Nanog) and neural crest (CD117) marker expression were detectable in all DPSCs examined.

### Oxidative stress biomarker detection in DPSCs

**Fig. 6:**
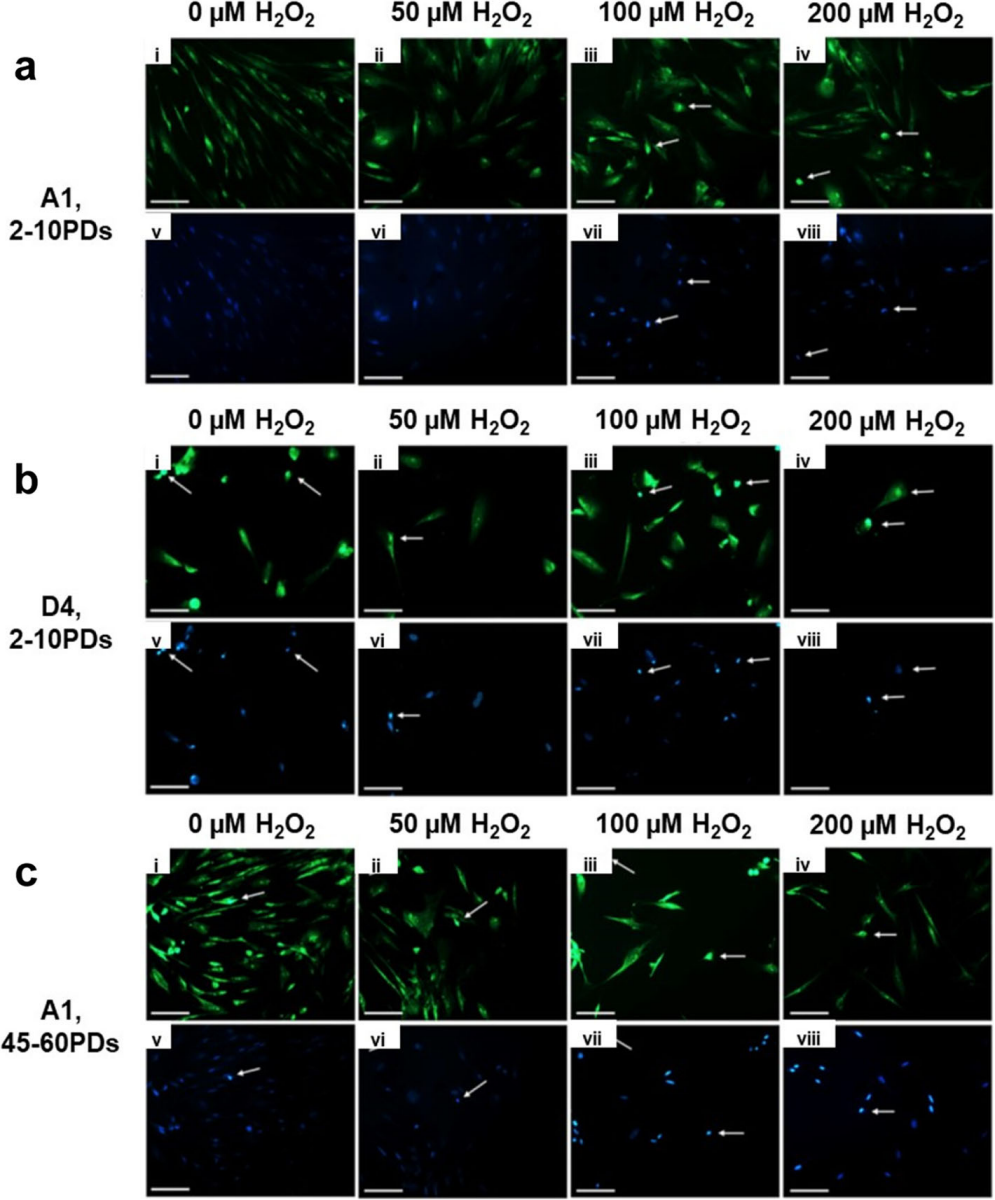
Oxidative DNA damage detection during extended DPSC culture with or without exogenous H_2_O_2_ (50–200 μM) treatment. Representative FITC (green, i–iv) and Hoechst nuclear stain (blue, v-viii) fluorescence microscopy images of 8-OHdG detection by immunocytochemistry, for **a** high proliferative sub-population, A1 (2–10PDs), **b** low proliferative sub-population, D4 (2–10PDs), and **c** high proliferative sub-population, A1 (45–60PDs). *N* = 3, scale bar 100 μm, × 200 magnification

As premature senescence is commonly associated with increased oxidative DNA and protein damage [20–22], we next investigated whether the high and low proliferative DPSC sub-populations also differed in their respective susceptibilities to oxidative stress-induced biomarker formation. Overall, oxidative DNA damage, in the form of 8-OHdG, showed marked variations in detection between high and low proliferative DPSC sub-populations (Fig. 6). High proliferative DPSC sub-population, A1, at 2–10PDs exhibited least positive nuclear DNA fluorescence detection for oxidative DNA damage overall, especially in untreated and 50 μM H_2_O_2_-treated cultures (Fig. 6a, i–ii). Although low-intensity cytoplasmic background staining was present, limited nuclear fluorescence was evident. However, increased nuclear DNA fluorescence staining intensities was identified for A1 at 2–10PDs, with 100–200 μM H_2_O_2_ (*arrowed*, Fig. 6a, iii-iv). There was also strong co-localization between oxidative DNA (fluorescein isothiocyanate, FITC) and Hoechst nuclear staining (*arrowed*, Fig. 6a, v-viii), thereby confirming the prominent nuclear localization of the oxidative DNA damage. In contrast, low proliferative DPSC sub-populations, A2 (Fig. S3), C3 (Fig. S3), and D4 (Fig. 6b) at 2–10PDs, all exhibited increased nuclear oxidative DNA damage, even in untreated controls (*arrowed*, i-iv and v-viii). High proliferative DPSC sub-population, A1, only exhibited similar nuclear FITC staining profiles to low proliferative DPSC sub-populations at 45–60PDs, both in untreated and H_2_O_2_-treated cultures (*arrowed*, Fig. 6c, i-iv and v-viii).

**Fig. 7:**
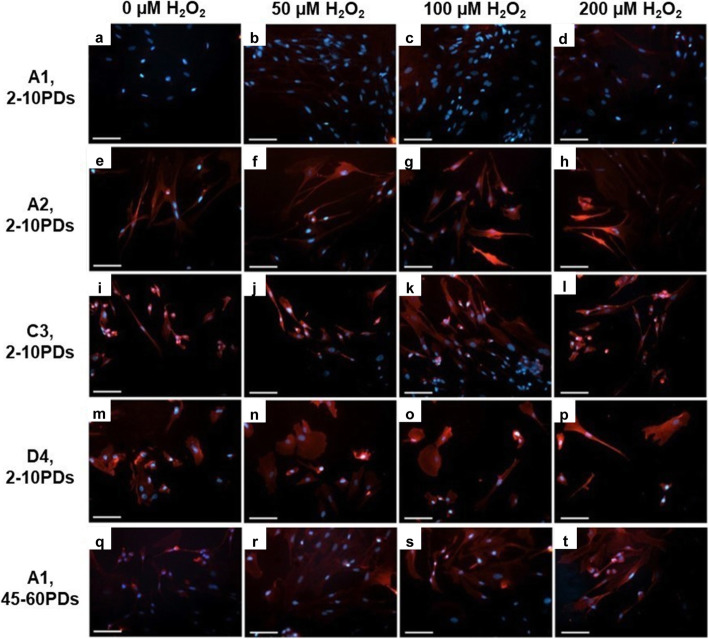
Oxidative protein damage detection during extended DPSC culture with or without exogenous H_2_O_2_ (50–200 μM) treatment. Representative merged TRITC (red) and Hoechst nuclear stain (blue) fluorescence microscopy images of protein carbonyl detection by immunocytochemistry, for **a–d** high proliferative sub-population, A1 (2–10PDs), **e–h** low proliferative sub-population, A2 (2–10PDs), **i–l** low proliferative sub-population, C3 (2–10PDs), **m–p** low proliferative sub-population, D4 (2–10PDs), and **q–t** high proliferative sub-population, A1 (45–60PDs). *N* = 3, scale bar 100 μm, × 200 magnification

Oxidative protein damage profiles, as protein carbonyl detection, also showed marked variations in detection between high and low proliferative DPSC sub-populations (Fig. 7). High proliferative DPSC sub-population, A1, at 2-10PDs exhibited least tetramethylrhodamine (TRITC) detection and oxidative protein damage overall, with minimal intensity cellular staining in both untreated and H_2_O_2_-treated cultures (Fig. 7a–d). In contrast, low proliferative DPSC sub-populations, A2, C3, and D4 at 2–10PDs, exhibited extensive intracellular detection of TRITC and oxidative protein damage, even in untreated controls (Fig. 7e–h, i–l, and m–p, respectively). High proliferative DPSC population, A1, only exhibited similar oxidative protein damage profiles to low proliferative DPSC sub-populations at 45–60PDs (Fig. 7q–t).

### SOD isoform gene expression and activities in DPSCs

**Fig. 8:**
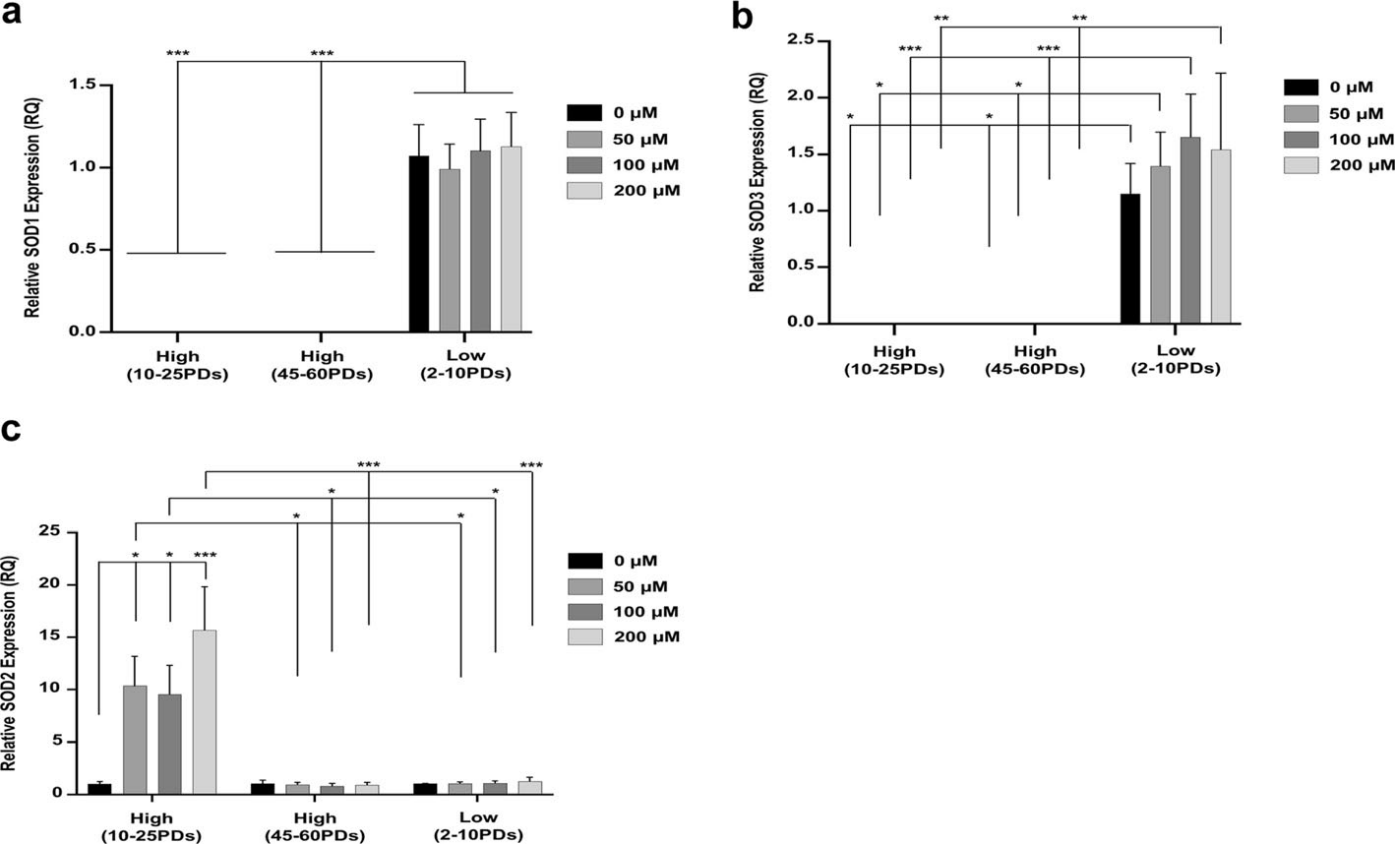
SOD isoform expression during extended DPSC culture with or without exogenous H_2_O_2_ (50–200 μM) treatment. **a–c** qRT-PCR analysis of SOD1, SOD3, and SOD2 gene expression by high proliferative (10–25PDs and 40–65PDs) and low proliferative (2–10PDs) DPSC sub-populations. Relative fold changes in enzymic antioxidant gene expression (RQ) were calculated using the 2^–ΔΔCt^ method, normalized versus an 18S rRNA housekeeping gene. *N* = 3, values in the graphs represent mean ± SEM, *p < 0.05, **p < 0.01, ***p < 0.001

Due to the well-established correlations between cellular susceptibilities to oxidative stress-induced damage, premature senescence and endogenous enzymic antioxidant levels [27–37], we next showed distinct differences in antioxidant gene expression, protein and activity profiles between high and low proliferative DPSC sub-populations. Firstly, qRT-PCR analysis demonstrated that SOD1 and SOD3 expression were undetectable in high proliferative DPSCs at 10–25PDs and 40–60PDs. However, significantly higher SOD1 and SOD3 expression were detectable in low proliferative DPSCs at 2–10PDs (all *p* < 0.001 and *p* < 0.001–0.05 respectively, Fig. 8a-b), but at relatively low levels with no significant increases in SOD expression with H_2_O_2_ treatment (*p* > 0.05). Despite no significant differences in SOD2 expression between high and low proliferative DPSCs at 10–25PDs and 2–10PDs, without H_2_O_2_ treatment (*p* > 0.05, Fig. 8c), high proliferative DPSCs demonstrated significantly higher (10–15 fold) inductions in SOD2 expression with H_2_O_2_ treatment, unlike low proliferative DPSCs (*p* < 0.001–0.05). However, high proliferative DPSCs at late PDs (45–60PDs) showed no significant differences in SOD2 expression (*p* > 0.05). Thus, H_2_O_2_-treated high proliferative DPSCs at 45–60PDs exhibited significantly lower SOD2 expression, than at 10–25PDs (*p* < 0.001–0.05).

**Fig. 9:**
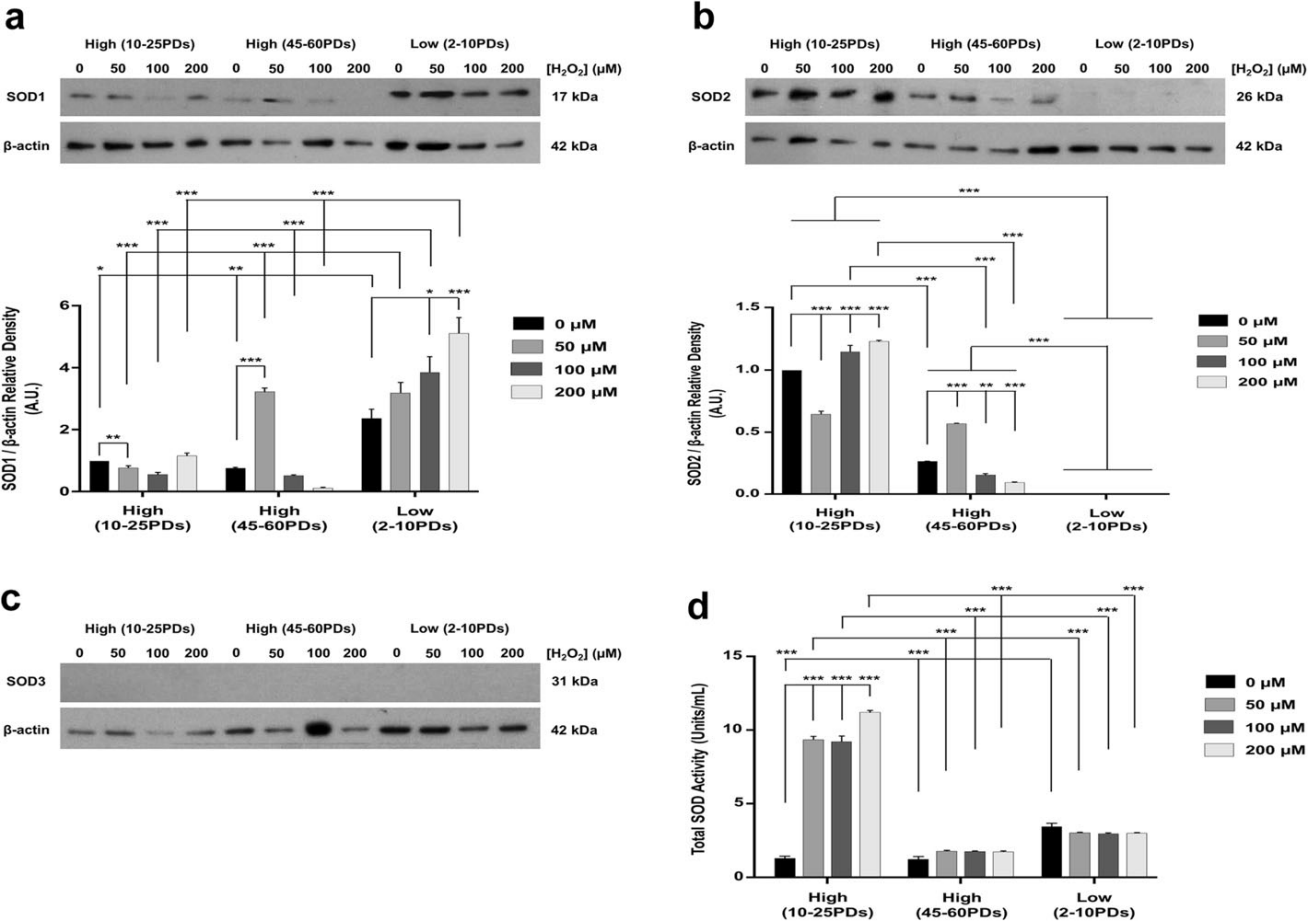
SOD isoform protein levels and total SOD activities during extended DPSC culture with or without exogenous H_2_O_2_ (50–200 μM) treatment. **a–c** Representative Western blot images and corresponding densitometric analysis of SOD1, SOD2, and SOD3 protein levels by high proliferative (10–25PDs and 40–65PDs) and low proliferative (2–10PDs) DPSC sub-populations. For all Western blots, images from one representative experiment of three are shown. Densitometry data was normalized versus β-actin loading controls, with values subsequently normalized versus untreated high proliferative DPSCs at early PDs (10–25PDs). A.U. = Arbitrary units. **d** Total SOD activities for high proliferative (10–25PDs and 40–65PDs) and low proliferative (2–10PDs) DPSC sub-populations. *N* = 3, values in the graphs represent mean ± SEM, *p < 0.05, **p < 0.01, ***p < 0.001

Based on these contrasting SOD expression profiles, we further examined whether such differences were evident at protein and activity levels. Western blot analysis demonstrated low levels of detectable SOD1 protein in high proliferative DPSCs at 10–25PDs, with no significant SOD1 inductions with increasing H_2_O_2_ treatment (*p* > 0.05), although reductions in SOD1 levels were evident with 50 μM H_2_O_2_ (*p* < 0.01, Fig. 9a). High proliferative DPSCs at 45–60PDs exhibited similar SOD1 levels to early PDs, although significant increases in SOD1 were identified with 50 μM H_2_O_2_ (*p* < 0.001). In contrast, untreated low proliferative DPSCs at 2–10PDs demonstrated significantly higher SOD1 levels, compared to high proliferative DPSCs (*p* < 0.01–0.05). Low proliferative DPSCs at 2–10PDs showed further significant increases in SOD1 levels with 100–200 μM H_2_O_2_ (*p* < 0.05 and *p* < 0.01, respectively). Thus, H_2_O_2_-treated low proliferative DPSCs at 2–10PDs possessed significantly higher SOD1 levels, versus high proliferative DPSCs at 10–25PDs and 45–60PDs (all *p* < 0.001).

SOD2 showed much higher levels of detectable protein in high proliferative DPSCs at 10–25PDs, compared to low proliferative DPSCs at 2–10PDs (*p* < 0.001, Fig. 9b). Although SOD2 levels significantly declined in high proliferative DPSCs at 10–25PDs with 50 μM H_2_O_2_ (*p* < 0.001), significant inductions in SOD2 levels were identified with 100–200 μM H_2_O_2_ (both *p* < 0.001). In contrast, SOD2 levels were virtually undetectable in low proliferative DPSCs at 2–10PDs (all *p* < 0.001 versus untreated and H_2_O_2_-treated high proliferative DPSCs). SOD2 levels in untreated high proliferative DPSCs at 45–60PDs also showed significant reductions, compared to early PDs (*p* < 0.001). Despite significant increases in SOD2 levels with 50 μM H_2_O_2_ (*p* < 0.001), significant decreases in SOD2 levels were shown with 100–200 μM H_2_O_2_, versus untreated controls (*p* < 0.01 and *p* < 0.001, respectively). However, despite such reductions, SOD2 levels in untreated and H_2_O_2_-treated high proliferative DPSCs at 45–60PDs remained significantly higher than low proliferative DPSCs at 2–10PDs (all *p* < 0.001). SOD3 protein was undetectable in all high and low proliferative DPSCs analyzed, irrespective of PDs and H_2_O_2_ treatments (Fig. 9c, *p* > 0.05).

SOD activities were significantly increased in low proliferative DPSCs at 2–10PDs, compared to high proliferative DPSCs at 10–25PDs and 45–60PDs, without H_2_O_2_ treatment (both *p* < 0.001, Fig. 9d). In contrast, high proliferative DPSCs at 10–25PDs demonstrated significantly increased SOD activities with H_2_O_2_ treatments (all *p* < 0.001), compared to untreated controls and to H_2_O_2_-treated low proliferative DPSCs, which failed to induce further increases in SOD activities. However, high proliferative DPSCs at 45–60PDs exhibited no increases in SOD activities with H_2_O_2_ treatment (*p* > 0.05 versus low proliferative DPSCs). Thus, H_2_O_2_-treated high proliferative DPSCs at 45–60PDs demonstrated significantly lower SOD activities, than at early PDs (all *p* < 0.001).

### Catalase gene expression and activities in DPSCs

**Fig. 10:**
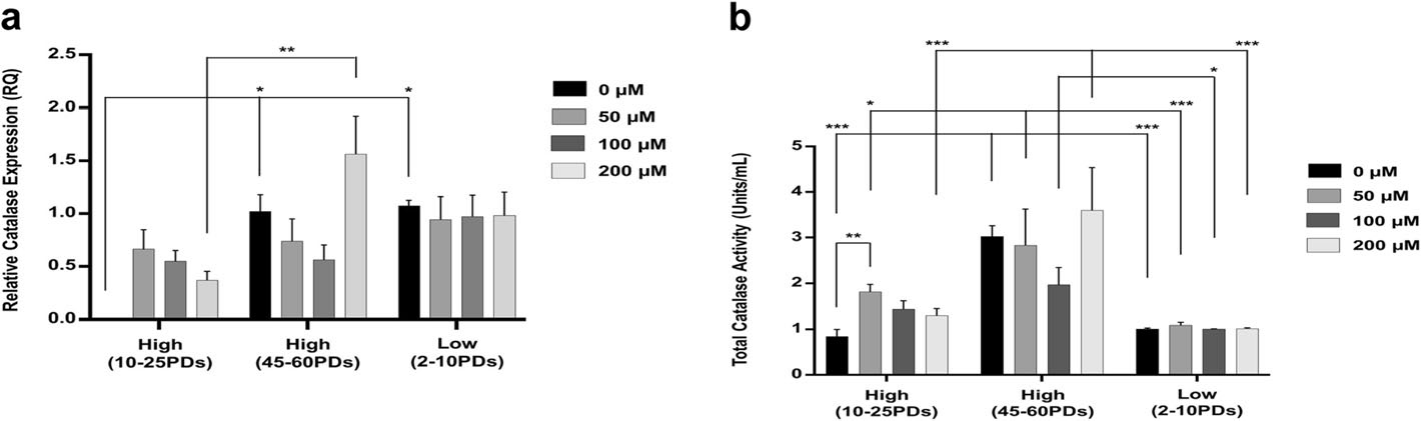
Catalase expression and activities during extended DPSC culture with or without exogenous H_2_O_2_ (50–200 μM) treatment. **a** qRT-PCR analysis of catalase gene expression by high proliferative (10–25PDs and 40–65PDs) and low proliferative (2–10PDs) DPSC sub-populations. Relative fold changes in enzymic antioxidant gene expression (RQ) were calculated using the 2^–ΔΔCt^ method, normalized versus an 18S rRNA housekeeping gene. **b** Total catalase activities for high proliferative (at 10–25PDs and 40–65PDs) and low proliferative (at 2–10PDs) DPSC sub-populations, treated as above. *N* = 3, values in the graphs represent mean ± SEM, *p < 0.05, **p < 0.01, ***p < 0.001

Catalase gene expression was maintained at relatively low levels in all high and low proliferative DPSCs analyzed (Fig. 10a). Although negligible basal catalase expression was determined in high proliferative DPSCs at 10–25PDs without H_2_O_2_ treatment, untreated low proliferative DPSCs at 2–10PDs exhibited higher expression (*p* < 0.05). However, neither high proliferative or low proliferative DPSCs demonstrated any further inductions in catalase expression with H_2_O_2_ treatment (all *p* > 0.05). High proliferative DPSCs at 45–60PDs without H_2_O_2_ treatment also demonstrated higher basal levels of catalase expression than at 10–25PDs (*p* < 0.05), although high proliferative DPSCs at 45–60PDs failed to promote further inductions in catalase expression with H_2_O_2_ treatment, except at 200 μM H_2_O_2_ (*p* < 0.01).

Although catalase activities were at similarly low levels in high and low proliferative DPSCs at 10–25PDs and 2–10PDs without H_2_O_2_ treatment (*p* > 0.05), significantly increased catalase activities were identified in high proliferative DPSCs at 45–60PDs without H_2_O_2_ treatment (both *p* < 0.001, Fig. 10b). High proliferative DPSCs at 10–25PDs only demonstrated significantly increased total catalase activities with 50 μM H_2_O_2_ (*p* < 0.01), versus untreated controls. However, equivalent catalase activities were shown between untreated and H_2_O_2_-treated high proliferative DPSCs at 45–60PDs (all *p* > 0.05 versus untreated controls), which were significantly higher than at early PDs with 50 μM and 200 μM H_2_O_2_ treatments (*p* < 0.05 and *p* < 0.001, respectively). Low proliferative DPSCs at 2–10PDs without H_2_O_2_ treatment exhibited equivalent catalase activities to untreated high proliferative DPSCs at 10–25PDs (*p* > 0.05), but were unable to induce further catalase activities with H_2_O_2_ treatment (*p* > 0.05 versus untreated low proliferative DPSCs). Therefore, catalase activities for H_2_O_2_-treated low proliferative DPSCs at 2–10PDs were significantly lower than high proliferative DPSCs at 40–65PDs, irrespective of H_2_O_2_ treatment (*p* < 0.001–0.05).

### Glutathione-related antioxidant gene expression and activities in DPSCs

**Fig. 11:**
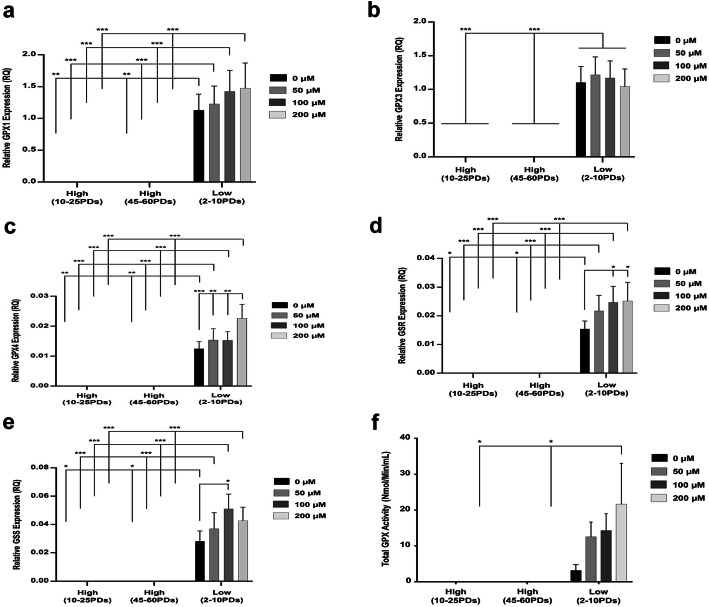
Glutathione-related antioxidant gene expression and activities during extended DPSC culture with or without exogenous H_2_O_2_ (50–200 μM) treatment. **a–c** qRT-PCR analysis of **a** GPX1, **b** GPX3, **c** GPX4, **d** GSR, and **e** GSS gene expression by high proliferative (10–25PDs and 40–65PDs) and low proliferative (2–10PDs) DPSC sub-populations. Relative fold changes in enzymic antioxidant gene expression (RQ) were calculated using the 2^–ΔΔCt^ method, normalized versus an 18S rRNA housekeeping gene. **f** Total GPX activities for high proliferative (at 10–25PDs and 40–65PDs) and low proliferative (at 2–10PDs) DPSC sub-populations. *N* = 3, values in the graphs represent mean ± SEM, *p < 0.05, **p < 0.01, ***p < 0.001

In terms of glutathione-related antioxidant gene expression, GPX2 and GPX5 were undetectable in all DPSCs analyzed (*data not shown*). GPX1, GPX3, GPX4, GSR, and GSS expression were also undetectable in high proliferative DPSCs at 10–25PDs and 40–60PDs. However, despite low proliferative DPSCs at 2–10PDs exhibiting significantly higher GPX1, GPX3, GPX4, GSR, and GSS expression (*p* < 0.001–0.05 versus high proliferative DPSCs), only relatively low levels of expression were detectable overall (Fig. 11a–e, respectively). Furthermore, both GPX1 and GPX3 demonstrated no significant inductions in expression by low proliferative DPSCs at 2–10PDs with H_2_O_2_ treatment (*p* > 0.05, Fig. 11a, b). However, significant increases in GPX4 expression were shown by low proliferative DPSCs at 2–10PDs with 200 μM H_2_O_2_, compared to their untreated and 50–100 μM H_2_O_2_-treated counterparts (*p* < 0.001–0.01, Fig. 11c). GSR and GSS expression were also significantly increased in low proliferative DPSCs at 2–10PDs, following treatment with 100–200 μM H_2_O_2_ and 100 μM H_2_O_2_, respectively (all *p* < 0.05, Fig. 11d, e). Consequently, total GPX activities were only detectable in low proliferative DPSCs at 2–10PDs at relatively low levels (*p* < 0.05 with 200 μM H_2_O_2_, versus high proliferative DPSCs at 10–25PDs and 45–60PDs, Fig. 11f), with no significant inductions in GPX activities with H_2_O_2_ treatment (all *p* > 0.05).

**Fig. 12:**
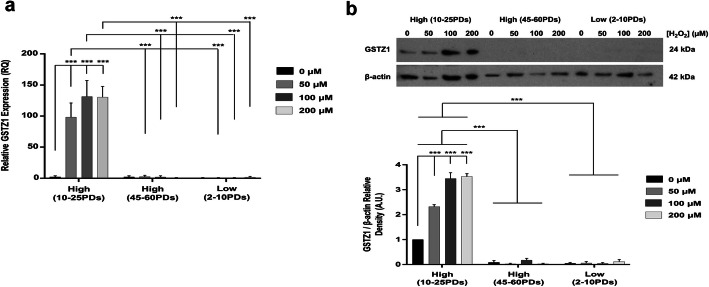
GSTZ1 gene expression and protein levels during extended DPSC culture with or without exogenous H_2_O_2_ (50–200 μM) treatment. **a** qRT-PCR analysis of GSTZ1 gene expression by high proliferative (10–25PDs and 40–65PDs) and low proliferative (2–10PDs) DPSC sub-populations. Relative fold changes in enzymic antioxidant gene expression (RQ) were calculated using the 2^–ΔΔCt^ method, normalized versus an 18S rRNA housekeeping gene. **b** Representative Western blot images and corresponding densitometric analysis of GSTZ1 protein levels by high proliferative (at 10–25PDs and 40–65PDs) and low proliferative (at 2–10PDs) DPSC sub-populations. For all Western blots, images from one representative experiment of three are shown. Densitometry data was normalized versus β-actin loading controls, with values subsequently normalized versus untreated high proliferative DPSCs at early PDs (10–25PDs). A.U. = Arbitrary units. *N* = 3, values in the graphs represent mean ± SEM, ***p < 0.001

Despite no significant differences in GSTZ1 expression between untreated high and low proliferative DPSCs at 10–25PDs and 2–10PDs (*p* > 0.05, Fig. 12a), only high proliferative DPSCs demonstrated significant inductions (100–125 fold) in GSTZ1 expression with H_2_O_2_ treatment (all *p* < 0.001 versus low proliferative DPSCs). However, no GSTZ1 inductions were evident in H_2_O_2_-treated high proliferative DPSCs at 45–60PDs, with similar levels to low proliferative DPSCs at 2–10PDs (*p* > 0.05). Therefore, H_2_O_2_-treated high proliferative DPSCs at 45–60PDs possessed significantly lower GSTZ1 expression, than at 10–25PDs (all *p* < 0.001). GSTZ1 further demonstrated significantly higher protein levels in high proliferative DPSCs at 10–25PDs, compared to the undetectable levels evident in low proliferative DPSCs at 2–10PDs, with further significant inductions in GSTZ1 levels with H_2_O_2_ treatment (all *p* < 0.001, Fig. 12b). Untreated and H_2_O_2_-treated high proliferative DPSCs at 45–60PDs also exhibited negligible GSTZ1 detection (all *p* < 0.001 versus high proliferative DPSCs at 10–25PDs).

## Discussion

Although DPSC susceptibility to replicative and oxidative stress-induced premature senescence has previously been recognized [12, 16, 42–44], this is the first study to demonstrate the existence of inherent differences in oxidative stress responses and differential enzymic antioxidant profiles between DPSC sub-populations with contrasting proliferative capabilities, which subsequently impact on their respective multi-potency, stemness, and other cellular characteristics [12, 13]. Despite the concept of DPSC proliferative and differentiation heterogeneity within dental pulp tissues being well-established [4–6], only recently have major variations in the proliferative potentials and susceptibilities to replicative senescence been confirmed between DPSC sub-populations, correlating with contrasting telomere lengths and the differentiation capabilities of individual populations [12]. Thus, it has been proposed that such high proliferative/multi-potent DPSCs are responsible for the extensive expansion potential of heterogeneous populations (> 120PDs) in vitro [4–6], as less proliferative, uni-potent DPSCs would be selectively lost during extended culture [12, 39]. However, as hTERT is unlikely to have a prominent role in maintaining telomere integrity in DPSCs [12, 14–16], we hypothesized that superior antioxidant capabilities contributed to the proliferative and multi-potency capabilities of high proliferative DPSC sub-populations.

As with our previous study confirming variations in replicative senescence susceptibilities between high (> 80PDs) and low (< 40PDs) proliferative DPSCs [12], present findings identified similar variations in the relative susceptibilities of DPSC sub-populations to oxidative stress-induced premature senescence. Although all DPSC sub-populations exhibited accelerated susceptibilities to premature senescence in a H_2_O_2_ dose-dependent manner, high proliferative DPSCs showed most resistance to H_2_O_2_-induced senescence, achieving 50–76PDs similar to untreated controls (> 80PDs). In contrast, low proliferative sub-populations collectively displayed accelerated premature senescence (4–32PDs with 50–200 μM H_2_O_2_), even in untreated controls (only reaching 11–34PDs). In support of their enhanced resistance to premature senescence, high proliferative DPSCs were further shown to possess fewer SA-β-galactosidase-positive cells and lacked the expression of p53 and p16^INK4a^, at PDs where low proliferative DPSCs demonstrated increased detection with p21^waf1^, particularly following H_2_O_2_ treatment [8]. MSC senescence is driven by tumor suppressors, such as p53, which promotes growth arrest by inducing p21^waf1^ expression, inhibiting G_1_-S phase progression. Therefore, p53 and p21^waf1^ regulate MSC expansion in an undifferentiated state. MSC senescence can also initiate p16^INK4a^ checkpoints, inducing senescence. Consequently, both p53 and p16^INK4a^ are regarded as the principal mediators of MSC senescence [16, 42, 43, 45]. As p21^waf1^ also maintains stem cell renewal [46, 47], this may explain the presence of early p21^waf1^ expression in all DPSCs analyzed. Nonetheless, contrasting p53 and p16^INK4a^ expression in high and low proliferative DPSC sub-populations further confirmed the early onset of premature senescence in low proliferative DPSCs. In agreement with previous reports, hTERT expression was undetectable in all DPSC sub-populations assessed [13–16].

A key reason identified to be responsible for contrasting proliferative responses and susceptibilities to replicative senescence were the mean telomere lengths between high and low proliferative DPSC sub-populations, with the superior telomere characteristics of high proliferative DPSCs permitting extended culture and protection from senescence [12]. In line with premature senescence occurring irrespective of extensive telomere shortening [8, 10, 17], all DPSCs largely retained their telomere length profiles during culture. Intriguingly, telomere lengths for high proliferative DPSC sub-population, A1, were the most influenced by extended culture and H_2_O_2_ treatment, implying that this sub-population also underwent a degree of telomere-dependent senescence during culture. Alternatively, prolonged culture in H_2_O_2_ can promote telomere shortening via oxidative damage and single-strand breaks [48, 49]. Although we can only speculate on the extent to which telomere-dependent/telomere-independent mechanisms contributed to telomere erosion and senescence in high proliferative sub-population, A1, it may be assumed that both mechanisms are involved.

Further studies assessed the impact of premature senescence on the expression of stem cell markers in high and low proliferative DPSC sub-populations. In line with previous findings, all DPSCs were positive for MSC markers, CD73, CD90, and CD105, and negative for hematopoietic stem cell marker, CD45 [12, 39]. Expression of MSC multi-potency markers, CD29, CD146, and CD271, were only evident in low proliferative DPSCs, as was the expression of stem cell differentiation regulator, CD166 [7, 12, 50, 51]. However, all DPSC sub-populations showed strong positive gene expression for self-renewal/multi-potency marker, BMI-1 [16]. In terms of embryonic/neural crest markers, Oct4 was absent in high proliferative DPSCs, but expressed in all low proliferative DPSC sub-populations. In contrast, SSEA4 and Slug were positively expressed in high proliferative DPSCs and most low proliferative DPSCs. Oct4 and SSEA4 maintain embryonic self-renewal and pluripotency [52, 53], while Oct4 and Slug are also implicated in promoting mesenchymal lineage commitment [53, 54]. In agreement with previous reports of declined stem cell marker expression, stemness and multi-potency characteristics in MSC populations during senescence [12, 16, 39, 44, 51], increasing H_2_O_2_ treatment and culture expansion reduced expression of CD73, CD105, SSEA4, and Slug in high proliferative DPSCs and Oct4 and CD271 in low proliferative DPSCs, potentially impacting on their stem cell and differentiation properties overall.

Having confirmed significant variations in DPSC sub-population susceptibility to premature senescence, high proliferative DPSCs were further shown to exhibit resistance to oxidative stress-induced biomolecular damage that gradually diminished with culture expansion. In contrast, low proliferative DPSC sub-populations showed much earlier oxidative stress biomarker detection, even without H_2_O_2_ treatment. Similar conclusions of elevated oxidative DNA and protein damage in low proliferative DPSC sub-populations have been reported by Raman Spectroscopy analysis [55]. Oxidative DNA damage is well-established to accompany cellular senescence [21, 56], which could contribute to the early-onset of p53, p21^waf1^, and p16^INK4a^ induction and increased premature senescence in low proliferative DPSC sub-populations [27–31, 36]. Oxidative protein damage, as particularly evident in low proliferative DPSCs, is also a well-documented occurrence during cellular senescence, due to oxidized protein modification and accumulation [22, 57].

The relative susceptibilities of DPSC sub-populations to oxidative damage and premature senescence suggested that such responses were related to contrasting antioxidant defense mechanisms between high and low proliferative DPSCs. High proliferative DPSCs were demonstrated to possess superior abilities to induce certain enzymic antioxidant expression and activities, compared to low proliferative DPSCs. The ability to upregulate antioxidant expression to counteract ROS is a fundamental concept of oxidative stress, including resistance to cellular senescence [27–37]. SOD profiles demonstrated distinct differences between high and low proliferative DPSCs, with low SOD1 and SOD3 levels particularly detectable in low proliferative DPSCs. In contrast, only high proliferative DPSCs at 10–25PDs demonstrated significantly induced SOD2 expression (10–15-fold) with H_2_O_2_ treatment. Such findings imply that SODs predominantly localized within cytosolic (SOD1) and extracellular (SOD3) regions do not contribute to the antioxidant status of high proliferative DPSCs, although induction of SOD1 and SOD3 expression in untreated and H_2_O_2_-treated low proliferative DPSCs imply that these sub-populations are experiencing oxidative stress [55]. The relatively high SOD2 induction in high proliferative DPSCs strongly suggests that SOD2 is a prominent mediator of antioxidant activity within these sub-populations [58]. As SOD1 and SOD2 are ubiquitously expressed by aerobic cells, the low SOD1 levels in high proliferative DPSCs is intriguing, although the absence of SOD3 can be explained by its more specific cellular expression profiles [19, 24]. Due to the limited SOD1 and SOD3 detection, it is likely that most SOD activity induced in H_2_O_2_-treated high proliferative DPSCs is accountable by upregulated SOD2 expression. In contrast, expression and protein analyses suggest that SOD1 is a principal contributor to SOD activities in low proliferative DPSCs.

Catalase profiles demonstrated higher expression and activities in low proliferative DPSCs, although only relatively low levels of catalase were detectable in DPSCs overall, even with H_2_O_2_ treatment. Similar catalase expression/activity profiles have been reported in high and low proliferative bone marrow-derived MSCs [58]. However, despite being a potent cytosolic H_2_O_2_ detoxifying antioxidant, catalase is particularly susceptible to downregulation and inactivation by ROS [59, 60], which may be responsible for the low catalase expression/activity levels detected. Thus, although catalase appears to have a relatively minor role in mediating antioxidant responses in high proliferative DPSCs, as with SOD1, induction of limited catalase expression in untreated and H_2_O_2_-treated low proliferative DPSCs may imply that these sub-populations are already experiencing elevated oxidative stress [55].

Analysis of glutathione-metabolizing enzymes demonstrated that GPX, GSR, and GSS expression and GPX activities were undetectable in high proliferative DPSCs. Similarly, although low proliferative DPSCs exhibited GPX1, GPX3, GPX4, GSR, and GSS expression and GPX4, GSR, and GSS induction with increasing H_2_O_2_ treatment, gene expression and GPX activities were relatively low overall. Such findings imply that glutathione-related enzymes are not major contributors to the antioxidant status of high proliferative DPSCs, although low proliferative DPSCs may be more reliant on these antioxidant mechanisms. However, only high proliferative DPSCs at 10–25PDs significantly induced the expression of GSTZ1 with H_2_O_2_ treatment (100–125-fold). While GPXs reduce glutathione (GSH) and GSR exerts antioxidant defenses through the decomposition of H_2_O_2_ and hydroperoxides [25, 26], GSTZs primarily detoxify xenobiotics and endobiotics within the cytosol and mitochondria [61].

Mitochondria are established as the principle cellular source of ROS during senescence [58, 62, 63]. Thus, mitochondrial-specific SOD2 is acknowledged as the primary enzymic antioxidant against oxidative damage within mitochondria and the prevention of cellular senescence [19, 24, 32, 44, 58]. Increased GSTZ1 expression, especially mitochondrial GSTZ1, has also been strongly associated with decreased human aging and prolonged longevity, partly due to reduced telomere shortening [64, 65]. Furthermore, GSTZ1^−/−^ mice possess alterations in mitochondrial ultrastructure, size, and activity, confirming the protective roles of GSTZ1 in mitochondria [61, 66]. Therefore, our findings imply that mitochondrial-derived ROS are significant mediators of oxidative damage and premature senescence in low proliferative DPSCs, whereas high proliferative DPSCs are more resistant due to significant adaptations in SOD2 and GSTZ1 expression, leading to the extended maintenance of proliferative, stem cell, multi-potency, and other cellular characteristics [12, 13]. However, the absence of SOD2 and GSTZ1 inductions with prolonged culture expansion suggest that these adaptive antioxidant mechanisms become defective, leading to increased susceptibility to oxidative damage and premature senescence. Similar findings have been reported in MSCs from other sources, with senescent cells exhibiting lower SOD, catalase, and GPX expression, resulting in reduced antioxidant status and overall increases in oxidative stress [34–37].

Despite the findings presented herein, a limitation of the present study is that it has compared oxidative stress-induced biomolecular damage and SOD2/GSTZ1 profiles within high proliferative DPSCs derived from only one patient (patient A). As high proliferative/multi-potent DPSCs are regarded as minority sub-populations within dental pulp tissues [7, 12, 55], current screening protocols are not completely efficient for the guaranteed isolation of high proliferative/multi-potent DPSC sub-populations from the dental pulp tissues of all patient donor teeth [67]. Consequently, low proliferative/uni-potent DPSCs are usually the predominant sub-populations isolated and as a result, high and low proliferative DPSC sub-populations were not compared from all collected patient teeth. Thus, the true nature of such high proliferative/multi-potent minority DPSC sub-populations advocates more detailed investigations to confirm their reproducible isolation, presence, and regenerative characteristics across a wider number of patient-matched high and low proliferative DPSCs from the same donor teeth, in order to fully establish the relationship between oxidative damage, SOD2 and GSTZ1 profiles and how these impact on the overall PD capabilities and multi-potent differentiation capabilities of individual DPSC sub-populations. Indeed, we can only speculate on the underlying reasons for such differences between high proliferative/multi-potent and low proliferative/uni-potent DPSC sub-populations at present, as intrinsic features, such as those associated with patient donor characteristics and/or their developmental origins and stem cell niche sources within dental pulp tissues could all be influential factors and warrant additional consideration [4, 5, 7, 12, 67].

## Conclusions

The present findings support the existence of inherent differences in enzymic antioxidant profiles and overall antioxidant status between high and low proliferative DPSCs, helping explain the contrasting susceptibilities to oxidative stress-induced biomolecular damage, premature senescence, and the heterogeneity between DPSC sub-populations overall. Identification of differential SOD2 and GSTZ1 induction profiles between high proliferative/multi-potent and low proliferative/uni-potent DPSC sub-populations enhances our understanding of DPSC biology and its inter-relationship with cellular senescence. The current findings imply that SOD2 and GSTZ1 profiles could provide additional characteristics to enhance the selective screening and isolation of superior quality, high proliferative/multi-potent DPSC sub-populations from whole dental pulp tissues. Ultimately, validation of SOD2 and GSTZ1 profile abilities to discriminate high proliferative/multi-potent DPSCs would aid their overall expansion, assessment, and more efficient stem cell manufacture and banking, thereby supporting the translational development of more effective DPSC-based therapies for clinical applications.

## Supplementary Information

Supplementary information accompanies this paper at https://doi.org/XXXXX.

**Additional file 1:.** Table S1. Primers used for RT-PCR analysis.

**Additional file 2:.** Table S2. Primers used for qRT-PCR analysis.

**Additional file 3: **Figure S1. Detection of SA-β-galactosidase staining and % positively stained cell calculations for low proliferative DPSC sub-populations, A2 (2-10PDs) and C3 (2-10PDs), during extended culture with or without exogenous H_2_O_2_ (50–200 μM) treatment. Scale bar 100 μm, × 10 magnification. *N* = 3, values represent the mean ± SEM. *p < 0.05, ***p < 0.001 versus untreated DPSC controls.

**Additional file 4:.** Figure S2. Detection of senescence-related marker (p53, p16^INK4a^, p21^waf1^, hTERT) expression in low proliferative DPSC sub-population, A2 (2-10PDs), during extended culture with or without exogenous H_2_O_2_ (50–200 μM) treatment. β-actin was used as the housekeeping gene. Right-hand lanes represent separate total human RNA positive controls, water and RT negative controls. bp = base pairs.

**Additional file 5: **Figure S3. Immunocytochemical detection of oxidative DNA damage in low proliferative DPSC sub-populations, A2 (2-10PDs) and C3 (2-10PDs), during extended culture with or without exogenous H_2_O_2_ (50–200 μM) treatment. Representative FITC (green, i-iv) fluorescence microscopy images of 8-OHdG marker detection (*arrowed*) and Hoechst nuclear stain (blue, v-viii). N = 3, scale bar 100 μm, × 200 magnification.

## Data Availability

The datasets used and/or analyzed during the current study are available from the corresponding author on reasonable request.

## Abbreviations

8-OHdG: 8-hydroxy-deoxy-guanosine
ANOVA: Analysis of variance
DIG: Digoxigenin
DPSC: Dental pulp stem cell
FCS: Fetal calf serum
FITC: Fluorescein isothiocyanate
GPX: Glutathione peroxidase
GSH: Reduced glutathione
GSR: Glutathione reductase
GSS: Glutathione synthetase
GST: Glutathione S-transferase
GSTZ1: Glutathione S-transferase ζ1
hTERT: Reverse transcriptase human telomerase catalytic subunit
αMEM: α-modified Minimum Essential Medium
MSC: Mesenchymal stem cell
PBS: Phosphate-buffered saline
PDs: Population doublings
pRb: Retinoblastoma protein
qRT-PCR: Quantitative real-time polymerase chain reaction
ROS: Reactive oxygen species
RQ: Relative fold changes in gene expression
RT-PCR: Reverse transcription polymerase chain reaction
SA-β-galactosidase: Senescence-associated-β-galactosidase
SASP: Senescence-associated secretory phenotype
SDS-PAGE: Sodium dodecyl sulfate-polyacrylamide gel electrophoresis
SEM: Standard error of mean
SOD: Superoxide dismutase
TBS: Tris-buffered saline
TRF: Telomere restriction fragment
TRITC: Tetramethylrhodamine

## References

[1] Ledesma-Martínez E, Mendoza-Núñez VM, Santiago-Osorio E (2016). Mesenchymal stem cells derived from dental pulp: a review. Stem Cells Int.

[2] Chalisserry EP, Nam SY, Park SH, Anil S (2017). Therapeutic potential of dental stem cells. J Tissue Eng.

[3] Anitua E, Troya M, Zalduendo M (2018). Progress in the use of dental pulp stem cells in regenerative medicine. Cytotherapy..

[4] Gronthos S, Mankani M, Brahim J, Robey PG, Shi S (2000). Postnatal human dental pulp stem cells (DPSCs) *in vitro* and *in vivo*. Proc Natl Acad Sci U S A.

[5] Gronthos S, Brahim J, Li W, Fisher LW, Cherman N, Boyde A, DenBesten P, Robey PG, Shi S (2002). Stem cell properties of human dental pulp stem cells. J Dent Res.

[6] Huang GT, Gronthos S, Shi S (2009). Mesenchymal stem cells derived from dental tissues vs. those from other sources: their biology and role in regenerative medicine. J Dent Res.

[7] Sloan AJ, Waddington RJ (2009). Dental pulp stem cells: what, where, how?. Int J Paediatr Dent.

[8] Campisi J, d'Adda di Fagagna F (2007). Cellular senescence: when bad things happen to good cells. Nat Rev Mol Cell Biol.

[9] Wagner W, Ho AD, Zenke M (2010). Different facets of aging in human mesenchymal stem cells. Tissue Eng Part B Rev.

[10] Li Y, Wu Q, Wang Y, Li L, Bu H, Bao J (2017). Senescence of mesenchymal stem cells (Review). Int J Mol Med.

[11] Malaquin N, Martinez A, Rodier F (2016). Keeping the senescence secretome under control: molecular reins on the senescence-associated secretory phenotype. Exp Gerontol.

[12] Alraies A, Alaidaroos NY, Waddington RJ, Moseley R, Sloan AJ (2017). Variation in human dental pulp stem cell ageing profiles reflect contrasting proliferative and regenerative capabilities. BMC Cell Biol.

[13] Alraies A, Waddington RJ, Sloan AJ, Moseley R (2020). Evaluation of dental pulp stem cell heterogeneity and behaviour in 3D type I collagen gels. Biomed Res Int.

[14] Flores I, Blasco MA (2010). The role of telomeres and telomerase in stem cell aging. FEBS Lett.

[15] Egbuniwe O, Idowu BD, Funes JM, Grant AD, Renton T, Di Silvio L (2011). P16/p53 expression and telomerase activity in immortalized human dental pulp cells. Cell Cycle.

[16] Mehrazarin S, Oh JE, Chung CL, Chen W, Kim RH, Shi S, Park NH, Kang MK (2011). Impaired odontogenic differentiation of senescent dental mesenchymal stem cells is associated with loss of Bmi-1 expression. J Endo.

[17] Denu RA, Hematti P (2016). Effects of oxidative stress on mesenchymal stem cell biology. Oxidative Med Cell Longev.

[18] Chaudhari P, Ye Z, Jang YY (2014). Roles of reactive oxygen species in the fate of stem cells. Antioxid Redox Signal.

[19] Sheshadri P, Kumar A (2016). Managing odds in stem cells: insights into the role of mitochondrial antioxidant enzyme MnSOD. Free Rad Res.

[20] Spiteller G (2001). Lipid peroxidation in aging and age-dependent diseases. Exp Gerontol.

[21] Evans MD, Dizdaroglu M, Cooke MS (2004). Oxidative DNA damage and disease: induction, repair and significance. Mutat Res.

[22] Davies MJ (2016). Protein oxidation and peroxidation. Biochem J.

[23] Kirkman HN, Gaetani GF (2007). Mammalian catalase: a venerable enzyme with new mysteries. Trends Biochem Sci.

[24] Fukai T, Ushio-Fukai M (2011). Superoxide dismutases: role in redox signaling, vascular function, and diseases. Antioxid Redox Signal.

[25] Board PG, Menon D (2013). Glutathione transferases, regulators of cellular metabolism and physiology. Biochim Biophys Acta.

[26] Deponte M (2013). Glutathione catalysis and the reaction mechanisms of glutathione-dependent enzymes. Biochim Biophys Acta.

[27] Lorenz M, Saretzki G, Sitte N, Metzkow S, von Zglinicki T (2001). BJ fibroblasts display high antioxidant capacity and slow telomere shortening independent of hTERT transfection. Free Rad Biol Med.

[28] Blander G, de Oliveira RM, Conboy CM, Haigis M, Guarente L (2003). Superoxide dismutase 1 knock-down induces senescence in human fibroblasts. J Biol Chem.

[29] Serra V, von Zglinicki T, Lorenz M, Saretzki G (2003). Extracellular superoxide dismutase is a major antioxidant in human fibroblasts and slows telomere shortening. J Biol Chem.

[30] Brown MF, Stuart JA (2007). Correlation of mitochondrial superoxide dismutase and DNA polymerase β in mammalian dermal fibroblasts with species maximal lifespan. Mech Ageing Dev.

[31] Hammad G, Legrain Y, Touat-Hamici Z, Duhieu S, Cornu D, Bulteau AL, Chavatte L (2018). Interplay between selenium levels and replicative senescence in WI-38 human fibroblasts: a proteomic approach. Antioxidants (Basel).

[32] He T, Peterson TE, Holmuhamedov EL, Terzic A, Caplice NM, Oberley LW, Katusic ZS (2004). Human endothelial progenitor cells tolerate oxidative stress due to intrinsically high expression of manganese superoxide dismutase. Arterioscler Thromb Vasc Biol.

[33] Valle-Prieto A, Congret PA (2010). Human mesenchymal stem cells efficiently manage oxidative stress. Stem Cells Dev.

[34] Ko E, Lee KY, Hwang DS (2012). Human umbilical cord blood-derived mesenchymal stem cells undergo cellular senescence in response to oxidative stress. Stem Cells Dev.

[35] Jeong SG, Cho GW (2015). Endogenous ROS levels are increased in replicative senescence in human bone marrow mesenchymal stromal cells. Biochem Biophys Res Commun.

[36] Yu J, Shi J, Zhang Y, Zhang Y, Huang Y, Chen Z, Yang J (2018). The replicative senescent mesenchymal stem/stromal cells defect in DNA damage response and anti-oxidative capacity. Int J Med Sci.

[37] Chen X, Wang L, Hou J, Li J, Chen L, Xia J, Wang Z, Xiao M, Wang Y (2019). Study on the dynamic biological characteristics of human bone marrow mesenchymal stem cell senescence. Stem Cells Int.

[38] Cristofalo VJ, Allen RG, Pignolo RJ, Martin BG, Beck JC (1998). Relationship between donor age and the replicative lifespan of human cells in culture: a re-evaluation. Proc Natl Acad Sci U S A.

[39] Lee CP, Colombo JS, Ayre WN, Sloan AJ, Waddington RJ (2015). Elucidating the cellular actions of demineralised dentine matrix extract on a clonal dental pulp stem cell population in orchestrating dental tissue repair. J Tiss Eng.

[40] Midgley AC, Morris G, Phillips AO, Steadman R (2016). 17β-estradiol ameliorates age-associated loss of fibroblast function by attenuating IFN-γ/STAT1-dependent miR-7 upregulation. Aging Cell.

[41] Livak KJ, Schmittgen TD (2001). Analysis of relative gene expression data using real-time quantitative PCR and the 2^–ΔΔCt^ method. Methods..

[42] Muthna D, Soukup T, Vavrova J, Mokry J, Cmielova J, Visek B, Jiroutova A, Havelek R, Suchanek J, Filip S (2010). Irradiation of adult human dental pulp stem cells provokes activation of p53, cell cycle arrest, and senescence but not apoptosis. Stem Cells Dev.

[43] Feng X, Xing J, Feng G, Huang D, Lu X, Liu S, Tan W, Li L, Gu Z (2014). p16^INK4A^ mediates age-related changes in mesenchymal stem cells derived from human dental pulp through the DNA damage and stress response. Mech Ageing Dev.

[44] Mas-Bargues C, Viña-Almunia J, Inglés M, Sanz-Ros J, Gambini J, Ibáñez-Cabellos JS, García-Giménez JL, Viña J, Borrás C (2017). Role of p16^INK4a^ and BMI-1 in oxidative stress-induced premature senescence in human dental pulp stem cells. Redox Biol.

[45] Shibata KR, Aoyama T, Shima Y, Fukiage K, Otsuka S, Fura M, Kohno Y, Ito K, Fujibayashi S, Neo M (2007). Expression of the p16^INK4A^ gene is associated closely with senescence of human mesenchymal stem cells and is potentially silenced by DNA methylation during *in vitro* expansion. Stem Cells.

[46] Kippin TE, Martens DJ, van der Kooy D (2005). p21 loss compromises the relative quiescence of forebrain stem cell proliferation leading to exhaustion of their proliferation capacity. Genes Dev.

[47] Ju Z, Choudhury AR, Rudolph KL (2007). A dual role of p21 in stem cell aging. Ann N Y Acad Sci.

[48] Duan J, Duan J, Zhang Z, Tong T (2005). Irreversible cellular senescence induced by prolonged exposure to H_2_O_2_ involves DNA-damage and -repair genes and telomere shortening. Int J Biochem Cell Biol.

[49] Brandl A, Meyer M, Bechmann V, Nerlich M, Angele P (2011). Oxidative stress induces senescence in human mesenchymal stem cells. Exp Cell Res.

[50] Kang CM, Kim H, Song JS, Choi BJ, Kim SO, Jung HS, Moon SJ, Choi HJ (2016). Genetic comparison of stemness of human umbilical cord and dental pulp. Stem Cells Int.

[51] Jin HJ, Kwon JH, Kim M, Bae YK, Choi SJ, Oh W, Yang YS, Jeon HB (2016). Downregulation of melanoma cell adhesion molecule (MCAM/CD146) accelerates cellular senescence in human umbilical cord blood-derived mesenchymal stem cells. Stem Cells Transl Med.

[52] Kawanabe N, Murata S, Fukushima H, Ishihara Y, Yanagita T, Yanagita E, Ono M, Kurosaka H, Kamioka H, Itoh T (2012). Stage-specific embryonic antigen-4 identifies human dental pulp stem cells. Exp Cell Res.

[53] Huang CE, Hu FW, Yu CH, Tsai LL, Lee TH, Chou MY, Yu CC (2014). Concurrent expression of Oct4 and Nanog maintains mesenchymal stem-like property of human dental pulp cells. Int J Mol Sci.

[54] Tang Y, Weiss SJ (2017). Snail/Slug-YAP/TAZ complexes cooperatively regulate mesenchymal stem cell function and bone formation. Cell Cycle.

[55] Alraies A, Canetta E, Waddington RJ, Moseley R, Sloan AJ (2019). Discrimination of dental pulp stem cell regenerative heterogeneity by single-cell Raman spectroscopy. Tissue Eng Part C Meth.

[56] Barnes RP, Fouquerel E, Opresko PL (2019). The impact of oxidative DNA damage and stress on telomere homeostasis. Mech Ageing Dev.

[57] Grune T, Merker K, Jung T, Sitte N, Davies KJ (2005). Protein oxidation and degradation during postmitotic senescence. Free Rad Biol Med..

[58] Bertolo A, Capossela S, Fränkl G, Baur M, Pötzel T, Stoyanov J (2017). Oxidative status predicts quality in human mesenchymal stem cells. Stem Cell Res Ther.

[59] Sadi G, Yılmaz Ö, Güray T (2008). Effect of vitamin C and lipoic acid on streptozotocin-induced diabetes gene expression: mRNA and protein expressions of cu–Zn SOD and catalase. Mol Cell Biochem.

[60] Waddington RJ, Alraies A, Colombo JS, Sloan AJ, Okazaki J, Moseley R (2011). Characterization of oxidative stress status during diabetic bone healing. Cells Tiss Organs.

[61] Board PG, Anders M (2011). Glutathione transferase ζ: discovery, polymorphic variants, catalysis, inactivation, and properties of Gstz1^−/−^ mice. Drug Metab Revs.

[62] Correia-Melo C, Marques FD, Anderson R, Hewitt G, Hewitt R, Cole J, Carroll BM, Miwa S, Birch J, Merz A, Rushton MD, Charles M, Jurk D, Tait SW, Czapiewski R, Greaves L, Nelson G, Bohlooly YM, Rodriguez-Cuenca S, Vidal-Puig A, Mann D, Saretzki G, Quarato G, Green DR, Adams PD, von Zglinicki T, Korolchuk VI, Passos JF (2016). Mitochondria are required for pro-ageing features of the senescent phenotype. EMBO J.

[63] Chapman J, Fielder E, Passos JF (2019). Mitochondrial dysfunction and cell senescence: deciphering a complex relationship. FEBS Lett.

[64] Di Cianni F, Campa D, Tallaro F, Rizzato C, De Rango F, Barale R, Passarino G, Canzian F, Gemignani F, Montesanto A (2013). MAP 3K7 and GSTZ1 are associated with human longevity: a two-stage case-control study using a multilocus genotyping. Age..

[65] Zhong G, James MO, Smeltz MG, Jahn SC, Langaee T, Simpson P, Stacpoole PW (2018). Age-related changes in expression and activity of human hepatic mitochondrial glutathione transferase ζ1. Drug Metab Disposit.

[66] Lim CE, Matthaei KI, Blackburn AC, Davis RP, Dahlstrom JE, Koina ME, Anders MW, Board PG (2004). Mice deficient in glutathione transferase ζ/maleylacetoacetate isomerase exhibit a range of pathological changes and elevated expression of α, μ, and π class glutathione transferases. Am J Pathol.

[67] Rodas-Junco BA, Villicaña C (2017). Dental pulp stem cells: Current advances in isolation, expansion and preservation. Tissue Eng Regen Med.

