# Supplementary material for: Differential SOD2 and GSTZ1 profiles contribute to contrasting dental pulp stem cell susceptibilities to oxidative damage and premature senescence
Source: Stem Cell Res Ther. 2021 Feb 17;12:142. doi: 10.1186/s13287-021-02209-9 (PMC7890809; doi:10.1186/s13287-021-02209-9)
Supplement: Supplementary file 1 — Additional file 1:. Table S1. Primers used for RT-PCR analysis. [file 13287_2021_2209_MOESM1_ESM.docx]

**Table S1.** Primers used for RT-PCR analysis.

| **Gene** | **Primer Sequence (5’-3’)** | **Length (bp)** |
| --- | --- | --- |
| p53 | F:5'- AGACCGGCGCACAGAGGAAG-3'  R:5'- CTTTTTGGACTTCAGGTGGC-3' | 280 |
| p21^waf1^ | F:5'- GGATGTCCGTCAGAACCCAT-3'  R:5'- CCCTCCAGTGGTGTCTCGGTG-3' | 165 |
| p16^INK4a^ | F:5'- CTTCCTGGACACGCTGGT-3'  R:5'- GCATGGTTACTGCCTCTGGT-3' | 162 |
| hTERT | F:5'- CGGAAGAGTGTCTGGAGCAA-3'  R:5'- GGATGAAGCGGAGTCTGG-3' | 145 |
| CD73 | F:5'-GTCGCGAACTTGCGCCTGGCCGCCAAG-3'  R:5'-TGCAGCGGCTGGCGTTGACGCACTTGC-3' | 352 |
| CD90 | F:5'- ATGAACCTGGCCATCAGCATCG-3'  R:5'- CACGAGGTGTTCTGAGCCAGCA-3' | 425 |
| CD105 | F:5'-GAAACAGTCCATTGTGACCTTCAG-3'  R: 5'-GATGGCAGCTCTGTGGTGTTGACC-3' | 425 |
| CD45 | F:5'-GTGACCCCTTACCTACTCACACCACTG-3'  R:5'-TAAGGTAGGCATCTGAGGTGTTCGCTG-3' | 455 |
| CD117 | F:5'- AAATCCATCCCCACACCCTG -3'  R:5'- CTTTTGTCGGCCTTGGTTGG -3' | 673 |
| CD146 | F:5'- ACAAGACCAAGATCCACAGCGAGT-3'  R:5'- ATGCACACAATCACAGCCACGATG-3' | 291 |
| CD166 | F:5'- TCATACCTTGCCGAGTTGACG-3'  R:5'- TCTGGTACTGGCCATCAATCC-3' | 330 |
| CD271 | F:5'- CTGCAAGCAGAACAAGCAAG-3'  R:5'- GGCCTCATGGGTAAAGGAGT-3' | 310 |
| BMI-1 | F:5'- CTGGTTGCCCATTGACAGCG -3'  R:5'- AAATCCCGGAAAGAGCAGCC-3' | 145 |
| Nanog | F:5'- TGCCTCACACGAGACTGTC-3'  R:5'- TGCTATTCTTCGGCCAGTTG-3' | 353 |
| Oct4 | F:5'- AGGAGTCGGGTGGAGAG-3'  R:5'- CGTTTGGCTGAATACCTTCC-3' | 250 |
| Slug | F:5'- GAGCATACAGCCCCATCACT-3'  R:5'- CTCCCCCGTGTGAGTTCTAA-3' | 479 |
| SSEA4 | F:5'- CAAAGAGGGGGACCCCTAGA-3'  R:5'- GACGGGGGAAATGTTACCGT-3' | 661 |
| β-actin | F:5'- AGGGCAGTGATCTCCTTCTGCATCCT-3'  R:5'- CCACACTGTGCCCATCTACGAGGGGT-3' | 480 |
