# Supplementary material for: Differential SOD2 and GSTZ1 profiles contribute to contrasting dental pulp stem cell susceptibilities to oxidative damage and premature senescence
Source: Stem Cell Res Ther. 2021 Feb 17;12:142. doi: 10.1186/s13287-021-02209-9 (PMC7890809; doi:10.1186/s13287-021-02209-9)
Supplement: Supplementary file 2 — Additional file 2:. Table S2. Primers used for qRT-PCR analysis. [file 13287_2021_2209_MOESM2_ESM.docx]

**Table S2.** Primers used for qRT-PCR analysis.

| **Gene** | **TaqMan^®^ Assay Gene IDs** |
| --- | --- |
| SOD1 | Hs00533490_m1 |
| SOD2 | Hs00167309_m1 |
| SOD3 | Hs04973910_s1 |
| CAT | Hs00156308_m1 |
| GPX1 | Hs00829989_Gh |
| GPX2 | Hs01591589_m1 |
| GPX3 | Hs01041668_m1 |
| GPX4 | Hs00989766_m1 |
| GPX5 | Hs00559733_m1 |
| GSR | Hs00167317_m1 |
| GSS | Hs00609286_m1 |
| GSTZ1 | Hs01041668_m1 |
| 18S rRNA | 4310893E |
