# Supplementary material for: Differential SOD2 and GSTZ1 profiles contribute to contrasting dental pulp stem cell susceptibilities to oxidative damage and premature senescence
Source: Stem Cell Res Ther. 2021 Feb 17;12:142. doi: 10.1186/s13287-021-02209-9 (PMC7890809; doi:10.1186/s13287-021-02209-9)
Supplement: Supplementary file 3 — Additional file 3: Figure S1. Detection of SA-β-galactosidase staining and % positively stained cell calculations for low proliferative DPSC sub-populations, A2 (2-10PDs) and C3 (2-10PDs), during extended culture with or without exogenous H2O2 (50–200 μM) treatment. Scale bar 100 μm, × 10 magnification. N = 3, values represent the mean ± SEM. *p < 0.05, ***p < 0.001 versus untreated DPSC controls. [file 13287_2021_2209_MOESM3_ESM.pptx]

## Slide 1
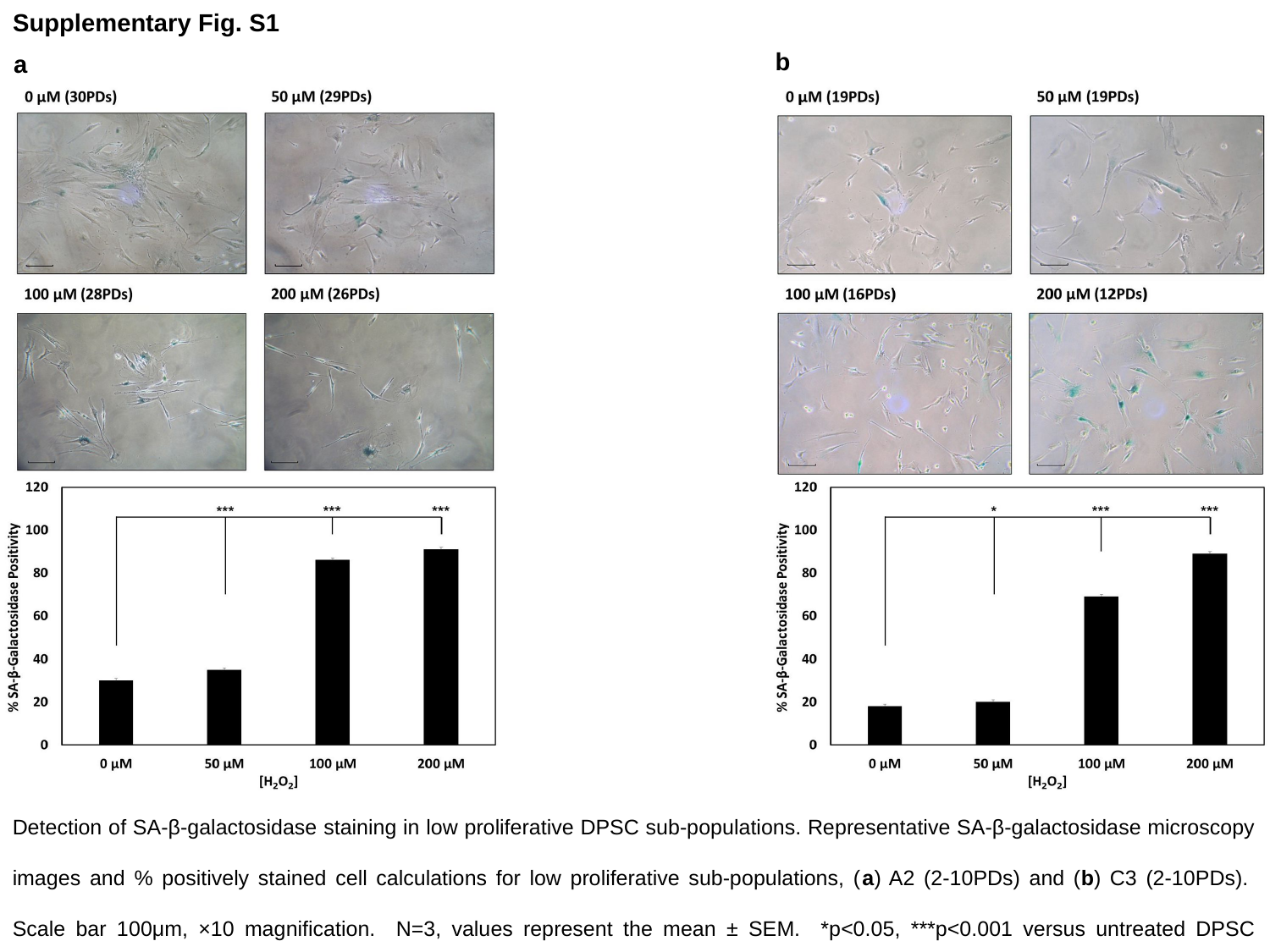

Supplementary Fig. S1
b
a
Detection of SA-β-galactosidase staining in low proliferative DPSC sub-populations. Representative SA-β-galactosidase microscopy images and % positively stained cell calculations for low proliferative sub-populations, (a) A2 (2-10PDs) and (b) C3 (2-10PDs). Scale bar 100μm, ×10 magnification. N=3, values represent the mean ± SEM. *p<0.05, ***p<0.001 versus untreated DPSC controls.
