# Supplementary material for: Differential SOD2 and GSTZ1 profiles contribute to contrasting dental pulp stem cell susceptibilities to oxidative damage and premature senescence
Source: Stem Cell Res Ther. 2021 Feb 17;12:142. doi: 10.1186/s13287-021-02209-9 (PMC7890809; doi:10.1186/s13287-021-02209-9)
Supplement: Supplementary file 4 — Additional file 4:. Figure S2. Detection of senescence-related marker (p53, p16INK4a, p21waf1, hTERT) expression in low proliferative DPSC sub-population, A2 (2-10PDs), during extended culture with or without exogenous H2O2 (50–200 μM) treatment. β-actin was used as the housekeeping gene. Right-hand lanes represent separate total human RNA positive controls, water and RT negative controls. bp = base pairs. [file 13287_2021_2209_MOESM4_ESM.pptx]

## Slide 1
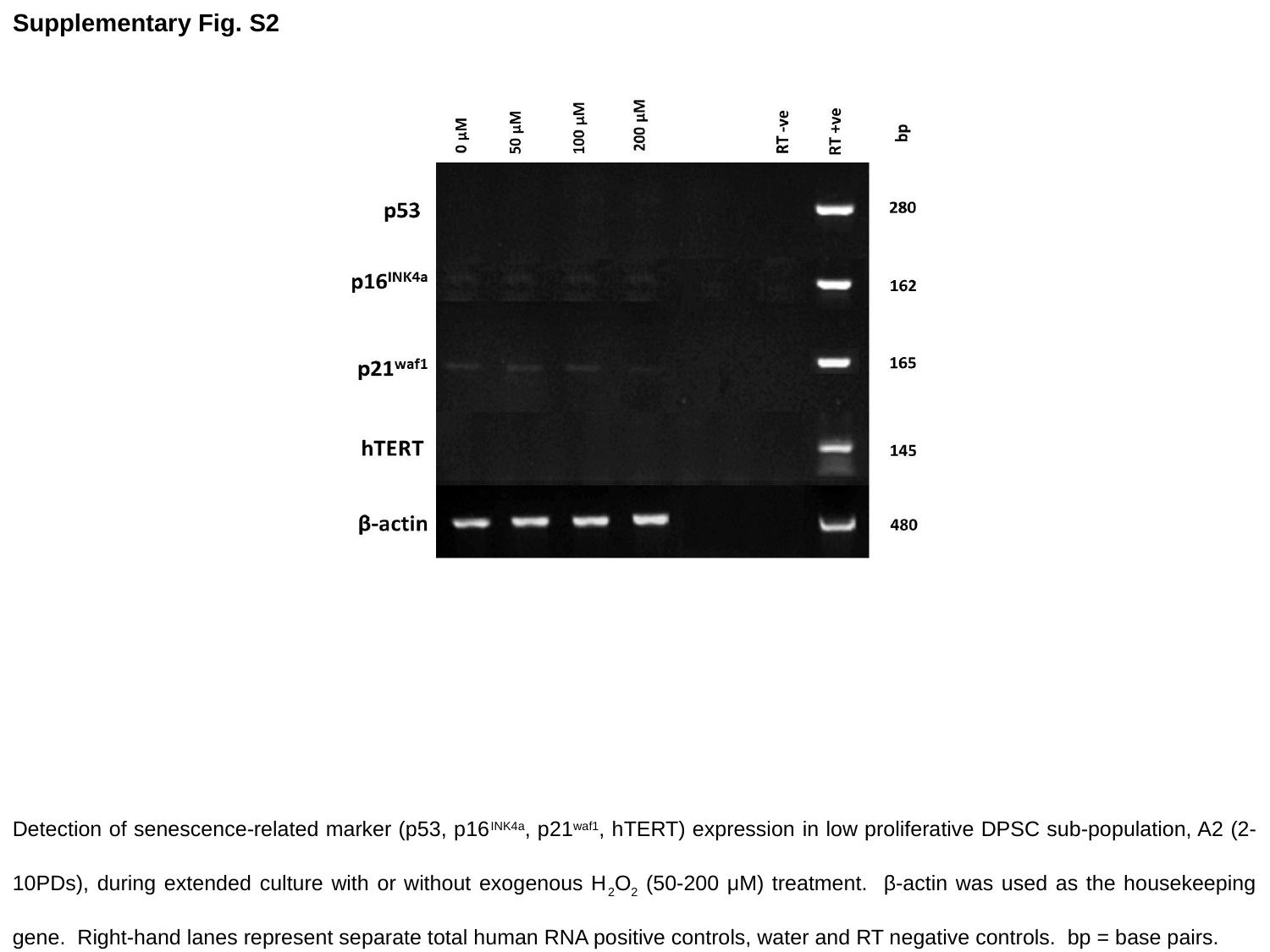

Supplementary Fig. S2
Detection of senescence-related marker (p53, p16INK4a, p21waf1, hTERT) expression in low proliferative DPSC sub-population, A2 (2-10PDs), during extended culture with or without exogenous H2O2 (50-200 μM) treatment. β-actin was used as the housekeeping gene. Right-hand lanes represent separate total human RNA positive controls, water and RT negative controls. bp = base pairs.
