# Supplementary material for: Differential SOD2 and GSTZ1 profiles contribute to contrasting dental pulp stem cell susceptibilities to oxidative damage and premature senescence
Source: Stem Cell Res Ther. 2021 Feb 17;12:142. doi: 10.1186/s13287-021-02209-9 (PMC7890809; doi:10.1186/s13287-021-02209-9)
Supplement: Supplementary file 5 — Additional file 5: Figure S3. Immunocytochemical detection of oxidative DNA damage in low proliferative DPSC sub-populations, A2 (2-10PDs) and C3 (2-10PDs), during extended culture with or without exogenous H2O2 (50–200 μM) treatment. Representative FITC (green, i-iv) fluorescence microscopy images of 8-OHdG marker detection (arrowed) and Hoechst nuclear stain (blue, v-viii). N = 3, scale bar 100 μm, × 200 magnification. [file 13287_2021_2209_MOESM5_ESM.pptx]

## Slide 1
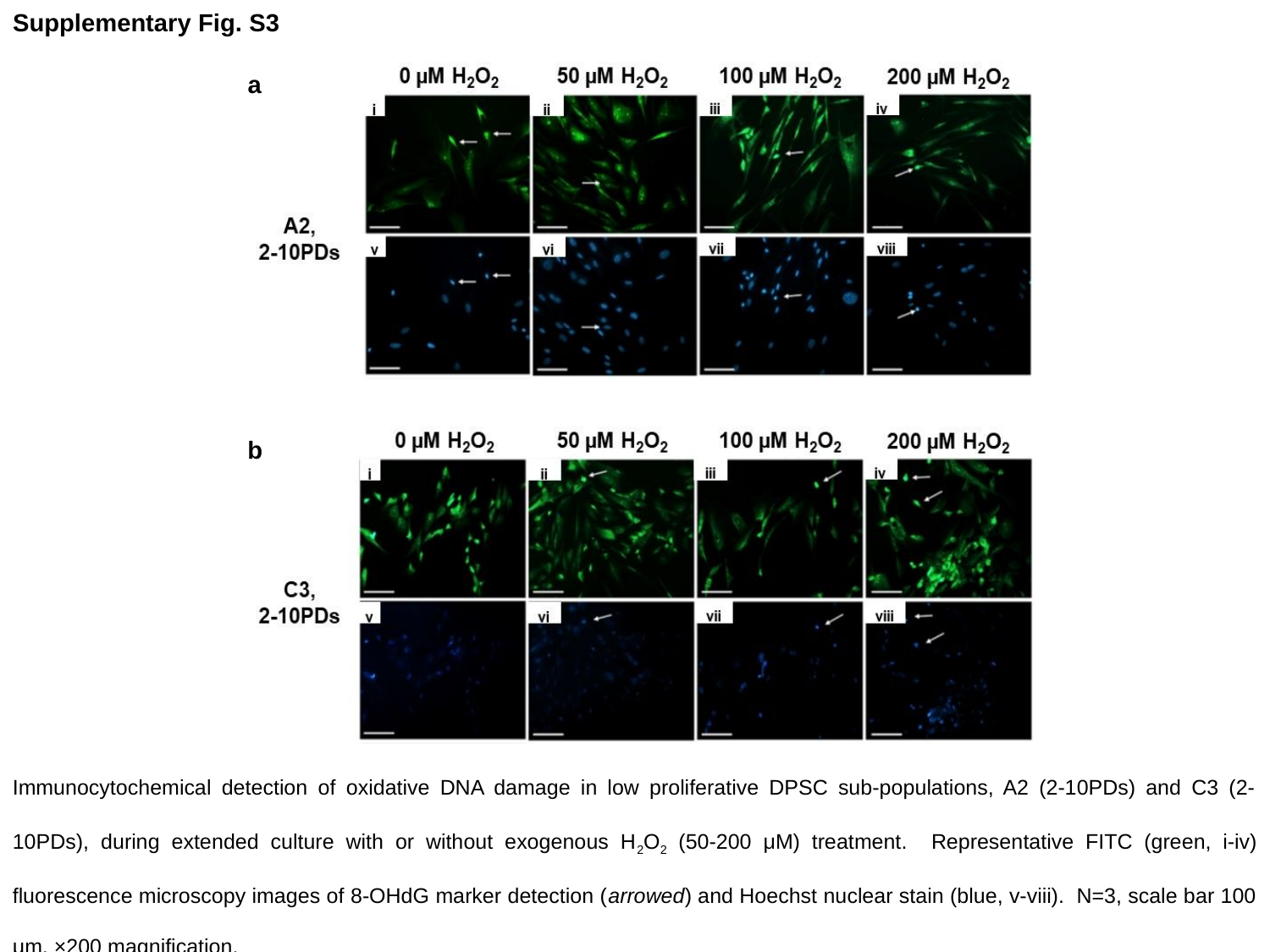

Supplementary Fig. S3
a
b
Immunocytochemical detection of oxidative DNA damage in low proliferative DPSC sub-populations, A2 (2-10PDs) and C3 (2-10PDs), during extended culture with or without exogenous H2O2 (50-200 μM) treatment. Representative FITC (green, i-iv) fluorescence microscopy images of 8-OHdG marker detection (arrowed) and Hoechst nuclear stain (blue, v-viii). N=3, scale bar 100 μm, ×200 magnification.
